# Electrospun Metal Oxide Nanofibers and Their Conductometric Gas Sensor Application. Part 2: Gas Sensors and Their Advantages and Limitations

**DOI:** 10.3390/nano11061555

**Published:** 2021-06-12

**Authors:** Ghenadii Korotcenkov

**Affiliations:** Department of Theoretical Physics, Moldova State University, 2009 Chisinau, Moldova; ghkoro@yahoo.com; Tel.: +373-60-642-109

**Keywords:** fabrication, nanofiber mat, single nanofiber, operation, performances, stability, optimization, advantages, limitations

## Abstract

Electrospun metal oxide nanofibers, due to their unique structural and electrical properties, are now being considered as materials with great potential for gas sensor applications. This critical review attempts to assess the feasibility of these perspectives. This article discusses approaches to the manufacture of nanofiber-based gas sensors, as well as the results of analysis of the performances of these sensors. A detailed analysis of the disadvantages that can limit the use of electrospinning technology in the development of gas sensors is also presented in this article. It also proposes some approaches to solving problems that limit the use of nanofiber-based gas sensors. Finally, the summary provides an insight into the future prospects of electrospinning technology for the development of gas sensors aimed for the gas sensor market.

## 1. Introduction

In recent years, noticeable interest has been shown in one-dimensional (1D) metal oxide nanomaterials [1,2,3,4,5,6,7,8,9,10] such as nanowires, nanobelts and nanotubes. Many authors also include metal oxide nanofibers (NFs) in this group of nanomaterials [11,12,13,14], although in nature, they differ from classical 1D nanomaterials. Unlike classical 1D nanomaterials, which are monocrystalline in nature, metal oxide nanofibers are amorphous or polycrystalline. Earlier in [15,16,17,18,19,20,21,22,23], it was shown that one of the most promising fields of application of metal oxide 1D nanomaterials is the development of conductometric gas sensors based on them. As it is followed from discussions presented in Part 1 of our article [24], metal oxide nanofibers are also a promising material for these applications, since a nanofiber mat, forming a gas-sensitive layer, is characterized by high porosity and a large surface-to-volume ratio [12,25,26]. In addition, the metal oxide crystallites in nanofibers can have an extremely small size [27]. According to generally accepted concepts [28,29,30,31,32], it is these parameters that should be possessed by materials capable of providing high sensitivity and a fast response of gas sensors. However, it is not clear how justified these expectations are from the use of nanofibers in conductometric gas sensors. For example, in [23], it was shown that 1D and 2D nanomaterials, despite the numerous advantages attributed to them [15,16,17,18,19,20,21,22], still do not find application in the development of gas sensors intended for the market.

In Part 1 [24], we declared that the second part of our article will be devoted to the analysis of nanofiber-based gas sensors and their advantages and limitations. This is exactly what is carried out in this article. In Part 2 of this article, approaches to the fabrication of gas sensors are considered, as well as the results of analysis of the performances of nanofiber-based conductometric gas sensors. It then provides a detailed analysis of the drawbacks that may limit the use of electrospinning technology in the development of gas sensors. Some approaches to solving these problems are also suggested in this part. Finally, the summary provides an insight into the future prospects of electrospinning applications for the development of conductometric gas sensors.

It is important to note here that the nature of gas-sensitive effects in nanofiber-based metal oxide gas sensors and conventional metal oxide gas sensors is identical, and therefore this topic will not be considered in this review. The mechanism of gas sensitivity, as well as the role of surface phenomena and structural factors in the sensor response of conductometric gas sensors, has been considered in sufficient detail in numerous reviews and books [28,29,30,31,32,33,34,35,36,37,38,39,40,41,42,43,44,45,46,47,48,49,50,51,52,53,54,55,56,57,58,59,60,61,62,63,64]. Most gas sensing mechanisms are based on the chemisorption of the target gas on the metal oxide surface or its interaction with chemisorbed oxygen. These processes are accompanied by a change in the surface charge, which determines the surface potential and thickness of the depleted layer, and, consequently, the conductivity of the gas-sensitive layer.

## 2. Fabrication of Gas Sensors Based on Metal Oxide Nanofibers

**Figure 1:**
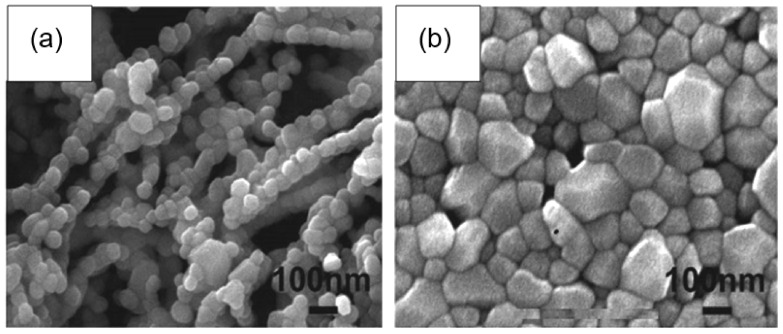
Morphology of WO_3_ (**a**) nanofibers and (**b**) films, fabricated using similar parameters. Deposition of W for 180 s, and oxidation at 700 °C in air for 2 h. Reprinted with permission from [27]. Copyright 2012 Royal Society of Chemistry.

As it was indicated before, nanofibers are not 1D structures, in the classical understanding, as metal oxide nanowires or nanobelts and nanotubes. However, research has shown that the features of the nanofiber configuration and the size factor play a positive role in the development of gas sensors based on nanofibers [12,26,65,66,67,68,69,70,71]. As mentioned above, metal oxide nanofibers, which usually have a diameter in the range of 50–1000 nm and a length from several micrometers to centimeters and meters, have many unique properties of gas-sensitive materials, such as a very large surface area per unit mass, high porosity and a small size of crystallites that form nanofibers. For example, gas-sensing materials formed from nanofibers usually have a porosity of ~70–90% [72]. The presence of large pores, along with small pores, that facilitate gas diffusion can also be attributed to the advantages of these materials. It is also important to note that, under equal conditions, the size of crystallites in a nanofiber will always be smaller (see Figure 1) than in a film formed by traditional technology [27]. Therefore, many developers believe that from this point of view, metal oxide nanofibers are an ideal candidate as sensing materials for gas sensors [12,25,73], since, unlike thin and thick films, gas-sensitive materials based on nanofibers can provide high gas permeability, even with the smallest crystallite size. Thin-film and thick-film technologies are deprived of this possibility, since with a decrease in the crystallite size, the pore size in the gas-sensitive material also decreases.

### 2.1. Sensors with Nanofiber Mat

**Figure 2:**
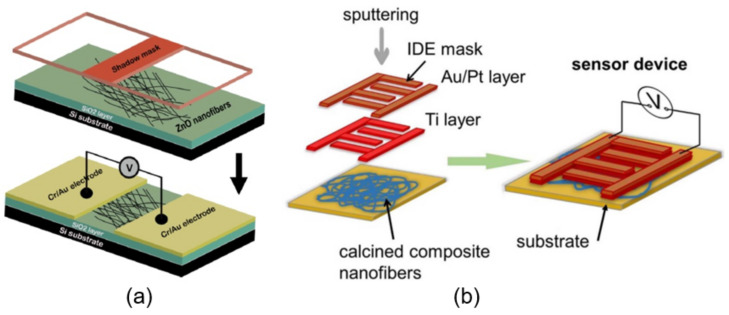
(**a**,**b**) Schematic illustrations of the fabrication process of metal oxide nanofiber-based sensor with (**a**) Au and (**b**) Ti-Au/Pt electrodes deposited on the nanofibers. (**a**) Reprinted from [78].; (**b**) Reprinted with permission from [79]. Copyright 2015 Elsevier.

The simplest method of manufacturing nanofiber-based sensors is shown in Figure 2. Generally, this process has three stages. First, the nanofibers are deposited on a fixed collector in a three-dimensional nonwoven membrane. Oxidized silicon wafers are usually used as a collector. Then, the deposited nanofibers are annealed, because of which an interconnected porous metal oxide structure is formed, and only then are metal contacts of various shapes, including interdigitated electrodes, applied to the formed mat of nanofibers [74,75,76]. Of course, the formation of a nanofiber membrane on a substrate with already applied electrodes is also possible [77].

As materials for the manufacture of electrodes, developers commonly use Au [78], Ag/Pd [80], Al [81], Ni [78], Ag [11], Ti [82] and Pt [79]. It would seem that the material of the electrodes should not affect the gas sensing characteristics. However, experiments carried out by Imran et al. [78], Wang et al. [81] and Batool et al. [82], who compared Au and Ni electrodes, Ag and Al electrodes and Ti, Au and Ni electrodes, respectively, showed that the electrode material can have a significant effect on the sensor parameters. Therefore, according to Imran et al. [78] and Wang et al. [81], sensors with Au and Ag electrodes exhibited improved sensing properties in comparison with sensors using Ni and Al electrodes. At the same time, according to Batool et al. [82], sensors with Au electrodes showed the worst characteristics in comparison with devices where Ti and Ni electrodes were used. Batool et al. [82] believe that such situation takes place due to the different porosities of electrode materials. According to Imran et al. [78], the work function of electrode materials plays a more important role. However, there are other factors that affect the performance of a sensor with different electrodes [28].

**Figure 3:**
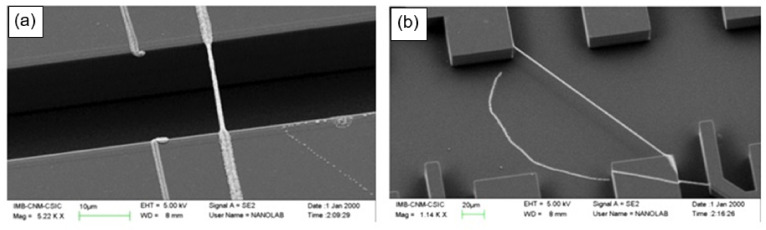
Electrospun ZnO nanobridges with lengths from (**a**) 20 µm to (**b**) 150 µm. A broken fiber is depicted to show that bridge formation is not a trivial process and the temperature ramp affects sintering. Reprinted from [83].

As for other experiences gained in the development and manufacture of nanofiber-based gas sensors, they can be formulated as follows:

- When manufacturing gas sensors using electrospun nanofibers suspended as bridges between contact areas, it is necessary to keep in mind that the heating rate during sintering of the metal oxide nanofibers is one of the most important factors. For example, Camargo et al. [83] showed that for manufacturing ZnO nanofiber-based bridges, the calcination of electrospun fibers at 600 °C should be carried out at a very low heating rate of ~5 °C/min. At a higher heating rate, the nanofibers were destroyed (see Figure 3). Camargo et al. [83] suggested that it is likely that the forces and mechanics involved during sintering require a slow temperature change.
- Formation of low-resistance contacts to nanofibers is a problem of manufacturing nanofiber-based gas sensors no less important than the formation of nanofibers with desired properties. Typically, contacts have increased resistance, which negatively affects the sensor performance. Camargo et al. [83], in order to form low-resistance contacts, used a focused ion beam (FIB)-assisted metal deposition. However, they themselves admit that FIB-assisted technology is a highly time-/money-demanding technique.
- In the absence of a proven sensor manufacturing technology, the role of the contact configuration increases significantly. Raible et al. [84] found that the contact of fibers with the top or bottom electrodes dramatically alters the response characteristics. In other words, for a better electrical contact, metal electrodes are preferably applied after nanofiber formation.

It should be noted that the problem of low-resistance contacts is an important problem for all conductometric gas sensors. If attention is not paid to this, a situation can occur where, under some conditions, the properties of the sensors will be controlled by inter-crystalline barriers, and in others, the sensor response will be controlled by the properties of the metal–nanofiber contact. In other words, sensors made even of the same gas-sensitive material can have fundamentally different characteristics. This is precisely the situation observed by Moon et al. [85] in a study of electrospun TiO_2_ nanofibers. They found that if In, Ag and Au form a low-resistance contact with TiO_2_ nanofibers, then a sputtered platinum electrode in contact with TiO_2_ forms a pronounced Schottky barrier with a potential barrier height of ~1.5 eV. For comparison, the height of the potential barrier at the boundary of TiO_2_ crystallites is ~0.7 eV. This means that the resistance of the Pt-TiO_2_ barrier even at T~200 °C is 10^9^ times greater than the resistance of inter-crystalline TiO_2_-TiO_2_ barriers. This is why Moon et al. [85] concluded that back-to-back Pt-TiO_2_ Schottky barriers, not TiO_2_-TiO_2_ grain boundaries, are responsible for the large resistance and conductometric response of TiO_2_ nanofiber-based sensors with Pt contacts to NO_2_. It is important to note that sensors with Pt contacts had the maximum sensitivity to NO_2_. Sensors with In and Au contacts had significantly lower sensor responses.

### 2.2. Single Nanofiber-Based Sensors

**Figure 4:**
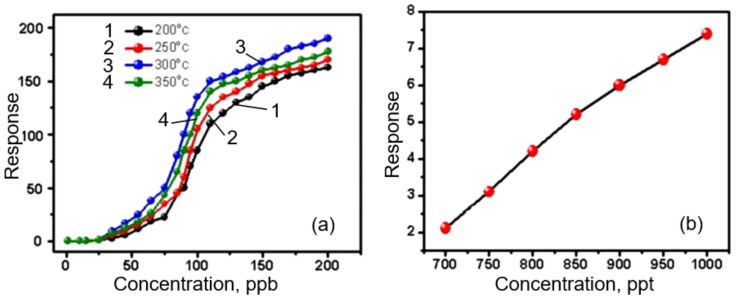
(**a**) Response of pure TiO_2_ single nanofiber triangular sample in the presence of CO gas as a function of concentration at 200−350 °C. (**b**) The response of TiO_2_:Au triangular samples in the presence of CO gas as a function of concentration in ppt range at 250 °C. Reprinted with permission from [86]. Copyright 2017 ACS.

Studies have shown that, as in the case of nanowires discussed in [23], individual nanofibers can also be used to develop gas sensors. For example, Nikfarjam et al. [86] fabricated individual TiO_2_ nanofiber-based sensors for detecting CO at concentrations as low as 30 ppb, with a rapid response and recovery time at *T*_oper_ = 250 °C (*t*_res_/*t*_rec_ = 3/4 s) (see Figure 4). There have been other attempts to develop single nanofiber-based gas sensors [83,84,87,88,89]. However, no significant results were obtained in these studies.

**Figure 5:**
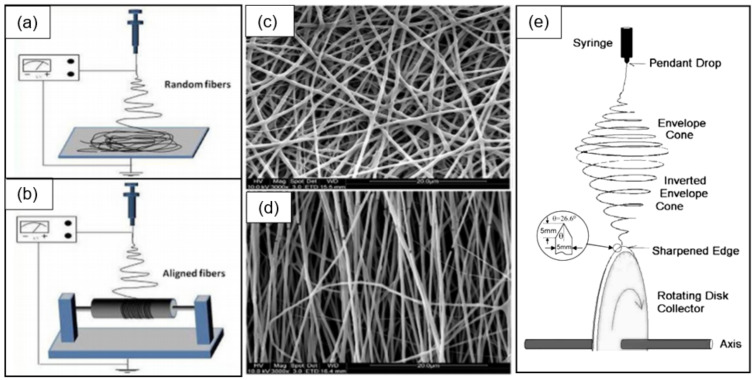
Different approaches to prepare electrospun nanofibers: (**a**) conventional approach, (**b**,**e**) obtaining aligned electrospun nanofibers (**b**) by a high-speed rotating drum and (**e**) by a sharp-edged rotating disk; (**c**,**d**) SEM images of (**c**) random and (**d**) aligned nanofibers. (**a-d**) Reprinted with permission from [96]. Copyrights 2013 Wiley; (**e**) Reprinted with permission from [97]. Copyright 2001 IOP.

It is clear that, as in the case of 1D nanowires, the formation of sensors based on individual nanofibers is associated with great difficulties due to the need to align, move and fix them in certain places [23]. However, it was found that the technology for fabricating individual nanofiber-based sensors can be significantly facilitated if nanofibers are deposited aligned and oriented during electrospinning. Studies carried out by various teams have shown that such conditions can indeed be realized (see Figure 5d). Recently, several approaches have been proposed to control the alignment of electrospun nanofibers [90,91]. One of the most common ways is to use the highly rotating drum as a collector [92,93] (Figure 5a). The properties and orientation of electrospun nanofibers in this case strongly depend on the drum rotation speed. A rotating disk with an extremely sharp edge can also be used instead of a rotating drum [94,95] (Figure 5e). However, since the edge of such a disk must be relatively sharp, this method has significant limitations if it is necessary to form well-aligned nanofibers over large areas.

**Figure 6:**
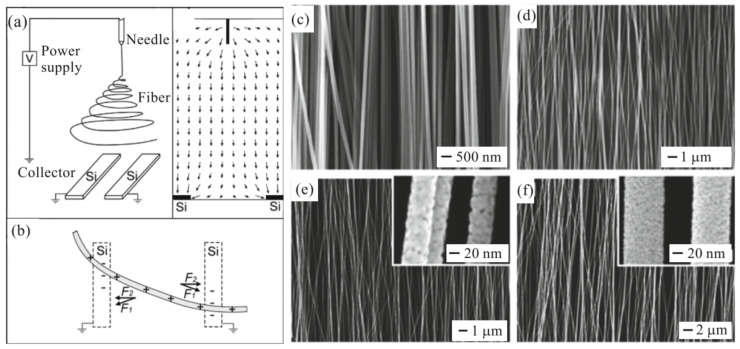
(**a**) Schematic illustration of the setup for collecting nanofibers as a uniaxially aligned array. The collector contains an insulating void, such as the air gap between two strips of silicon wafers. (**b**) Electrostatic force analysis of a charged nanofiber spanning across two silicon strips. The orientation of the nanofiber is mainly controlled by the stretching force originating from the attractive electrostatic forces. (**c**–**f**) SEM images of uniaxially aligned nanofibers made of (**c**) carbon, (**d**) anatase TiO_2_, (**e**) NiFe_2_O_4_ and (**f**) TiO_2_/PVP. Reprinted with permission from [101]. Copyright 2003 American Chemical Society.

**Figure 7:**
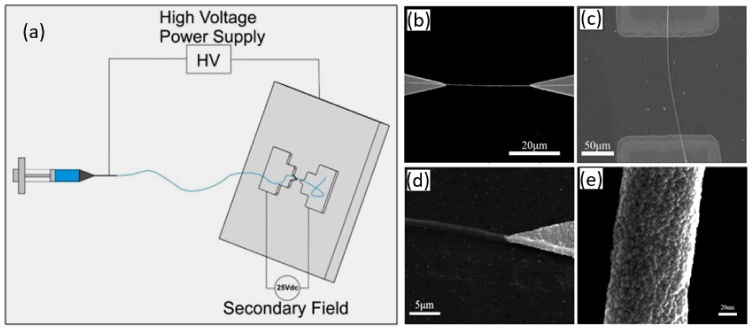
(**a**) Schematic representation of different fields during the electrospinning process. (**b**–**e**) FE-SEM images of (**b**) aligned pure single nanofiber on a triangular-type electrode, (**c**) aligned pure TiO_2_ single nanofiber on a rectangular-type electrode, (**d**) aligned fiber (before thermal treatment) on the tip of a triangular electrode and (**e**) TEM image of a pure TiO_2_ nanofiber. (**a**) Reprinted with permission from [86]. Copyrights 2017 ACS; (**b**–**e**) Adapted with permission from [141]. Copyright 2015 Elsevier.

There are several other methods for aligning and ordering nanofibers by the electrospinning method [89,90,98,99]. For example, there is magnetic field-assisted electrospinning [100]. However, the most progressive is still the method proposed by Li et al. [101,102]. Li et al. [101,102] demonstrated that nanofibers can be uniaxially aligned by introducing insulating gaps into conductive collectors (Figure 6). The insulating gap can be air, quartz, polystyrene or any other insulating material. The gap width can vary from hundreds of micrometers to several centimeters. According to Li et al. [102], fibers are pulled perpendicular to the edges of the gap due to electrostatic forces from two sources: a strong external field (F1) between the spinneret and the collector and repulsion between adjacent charged fibers (F2). SEM images of ceramic nanofibers made by this method are shown in Figure 6c–f. It is seen that the proposed method makes it possible to form well-aligned arrays of ceramic nanofibers. Single nanofibers deposited in controlled directions were also formed using this method. Li et al. [103] also showed that by changing the configuration of the electrodes, various structured structures of electrospun nanofibers can be obtained. The interdigital electrode can also be used to form oriented nanofibers. Ke et al. [104] found that by varying the configurations of the interdigital electrode, it is possible to obtain parallel electrospun nanofibers of different lengths. The main advantage of the methods developed by Li et al. [101,102] and Ke et al. [104] is that these methods allow the direct integration of nanofibers with controlled configurations into the electrode system used in the manufacture of gas sensors. Undoubtedly, this approach can significantly simplify the technology of manufacturing devices based on individual nanofibers. This very method was used to fabricate TiO_2_ nanofiber-based gas sensors, which were considered earlier in [86]. The only difference from the previously discussed methods is the use of a secondary field (Figure 7a). SEM images of a single aligned nanofiber used for gas sensor fabrication are shown in Figure 7b–e.

## 3. Performances of Gas Sensors Based on Metal Oxide Nanofibers

### 3.1. General Consideration

To date, there have been many attempts (listed in Table 1) to construct ultrasensitive gas sensors to detect NH_3_, H_2_S, CO, NO_2_, O_2_, CO_2_ and vapors of organic compounds (VOCs), such as CH_3_OH, C_2_H_5_OH, C_5_H_10_C_l2_, C_6_H_5_CH_3_ and C_4_H_8_O, with improved detection limits using nanofibrous membranes as sensing structures [12,27,87,105,106,107,108,109,110,111,112,113,114,115,116,117,118,119,120,121,122,123,124,125,126,127,128,129,130,131,132,133,134,135,136,137]. For example, Choi et al. [138] developed ZnO nanofiber-based NO_2_ sensors and compared them to conventional thin-film ZnO-based gas sensors. They found that the response of nanofiber-based NO_2_ sensors was higher and faster. Zhang et al. [139] reached the same conclusion when analyzing WO_3_ nanofiber-based NO_2_ gas sensors, and Du et al. [140] after considering the parameters of In_2_O_3_-based NH_3_ sensors.

It is important to note that reduced response and recovery times are a great advantage of nanofiber sensors, as a slow response and recovery were one of the major disadvantages of conventional gas sensors. Undoubtedly, this situation is due to the large surface area and high porosity of sensitive materials based on nanofibers. The extremely high porosity is the main advantage of these sensors, which show very good operating characteristics (excellent and fast response) compared to conventional sensor materials. This unique morphology facilitates the efficient penetration of the target gas into the porous ceramic layer, which is believed to be the main reason for the exceptionally high gas sensitivity of metal oxide gas sensors manufactured by this method [142]. Unlike conventional thin- and thick-film technologies, which produce mesoporous granular layers with densely packed nanoparticles that cause poor gas transfer, electrospinning sensors exhibit a bimodal pore size distribution, including both small and large pores that enhance gas transport and improve the conductometric response of these layers [143].

**Figure 8:**
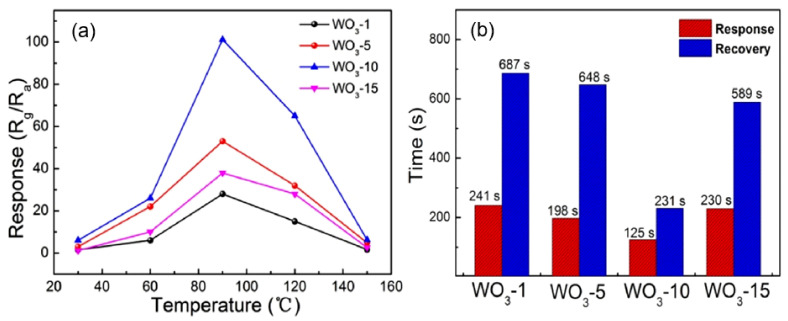
(**a**) Response–temperature curves of the four kinds of electrospun WO_3_-based sensors to 3 ppm NO_2_ at various temperatures; (**b**) the corresponding response and recovery times of all sensors to NO_2_ listed in Table 2. *T*_oper_ = 90 °C. Reprinted with permission from [139]. Copyright 2021 Elsevier.

The explanation of the gas sensing effect in metal oxide nanofibers can be carried out within the framework of the approaches developed for traditional gas sensors based on metal oxides [144]. Moreover, it was found that the gas sensing characteristics of sensors based on nanofibers made of metal oxides obey the same regularities as conventional metal oxide gas sensors discussed in [28,145,146]. This means that, as in the case of conventional gas sensors, nanofiber-based sensors with a smaller grain size, smaller crystallite size, higher porosity and larger surface area have the maximum conductometric response [36,147,148,149,150]. This conclusion is well illustrated in Figure 8. For example, Zhang et al. [139] showed that the maximum sensitivity and minimum response time were possessed by WO_3_ nanofiber-based sensors, which are characterized by the maximum surface area, minimum crystallite size and maximum pore diameter in nanofibers (see Table 2). A solution of ammonium metatungstate hydrate-PVP-deionized water was used for electrospinning. The WO_3_ nanofibers were obtained by annealing the nanofiber precursors at 550 °C for 2 h with different heating rates. To obtain nanofibers with different porosities, Zhang et al. [139] used annealing at different rates, varying from 1 to 15 °C/min.

**Table 1:** Metal oxide nanofiber-based conductometric gas sensors and their performances.

Material	Diameter, nm	Analyte Gas	C., ppm	T, ^o^C	Response	Res/Rec Time	Ref.
TiO_2_	120–200	NO_2_	50	450	30	2–4 min/20 s	[151]
WO_3_	100		0.4	75	12	33 min/38 min	[152]
SnO_2_	200–400		50	185	368	400 s/200 s	[153]
SnO_2_	300–500		2	300	81	55 s/5 min	[154]
CeO_2_	380	O_2_	100	800	1.4	30 s/-	[155]
La_0_._67_Sr_0_._33_MnO_3_	~126		100	800	1.1	53 s/-	[156]
ZnO	250	H_2_	10	350	109	-	[157]
TiO_2_	80		125	275	~6	12 s/22 s	[141]
WO_3_	200	NH_3_	100	200	6	1 s/5 s	[153]
ZnO	95–130		100	200	~20	-	[147]
TiO_2_	400–500	CO	25	200	~4	32–86 s/84–109 s	[158]
SnO_2_	200–400		500	300	~4	260 s/15 min	[143]
ZnO	35–150		2	200	1.5	168–237 s/270–350 s	[159]
In_2_O_3_	100		100	300	~5	-	[160]
WO_3_	275	Acetone	50	270	56	6–13 s/4–9 s	[161]
ZnO	145		1	220	7	12–17 s/11–23 s	[162]
In_2_O_3_	250–310		5	300	151	5 s/2 s	[163]
α-Fe_2_O_3_	150–280	Ethanol	100	300	2	3 s/5 s	[164]
SnO_2_	100		10	330	5	13 s/13.9 s	[105]
In_2_O_3_	160–200		1500	300	379	1 s/5 s	[165]
In_2_O_3_	30–100		30	220	~4	6 s/10 s	[166]
Co_3_O_4_	100–200		100	301	51	8–23 s/59–3 s	[167]
ZnO	500–600	DMF	100	RT	13	32 s/17 s	[168]
In_2_O_3_	150–200		100	340	3	18 s/17 s	[169]

**Table 2:** Parameters of electrospun WO_3_ nanofibers, used for fabrication of NO_2_ gas sensors.

Sample	Heating Rate, °C/min	Surface Area, m^2^/g	Pore Size, nm	Crystallite Size, nm
WO_3_-1	1	10.7	11.2	26.5
WO_3_-5	5	14.4	17.5	20.9
WO_3_ 10	10	16.4	30.6	16.8
WO_3_-15	15	12.8	18.5	16.1

*Source*: data extracted from [139].

**Figure 9:**
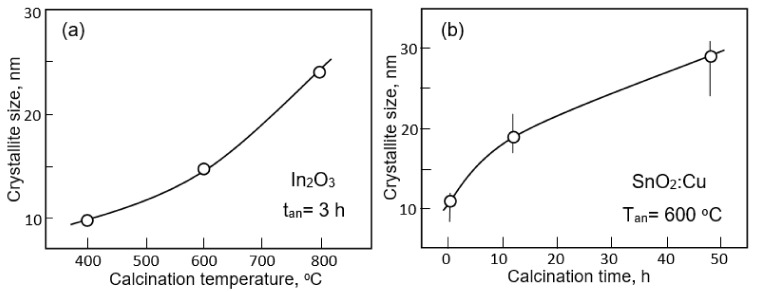
Influence of annealing (**a**) temperature (400–800 °C) and (**b**) time (0.5–48 h) on the crystallite size in (**a**) In_2_O_3_ and (**b**) SnO_2_:Cu nanofibers synthesized by electrospinning. (**a**) Data extracted from [171]; (**b**) Reprinted from [170].

As it was shown earlier, the crystallite size in nanofibers is usually controlled via adjusting the calcination temperature and time [170,171]. The higher the temperature and the longer the annealing time, the larger the crystallite size (see Figure 9). That is why, usually, the maximum sensor effect is observed at the minimum calcination temperature (see Figure 9a). However, it should be borne in mind that the lower the annealing temperature and the smaller the crystallite size, the more pronounced the temporal and thermal instability of the sensor parameters [55]. Therefore, when choosing calcination modes, a compromise between sensitivity and stability has to be found. The crystallite size can also be controlled by the solution composition and polymer content [106,172]. As a rule, an increase in the concentration of a precursor in an electrospinning solution is accompanied by an increase in the crystallite size of metal oxides (read Part 1 [24]). The diameter of nanofibers also has a strong influence on the crystallite size. The larger the nanofiber diameter, the larger the size of the metal oxide crystallites that are formed during the calcination process. To achieve the minimum diameter of nanofibers, the recommendations proposed earlier can be used.

### 3.2. Hollow Nanofiber-Based Gas Sensors

**Figure 10:**
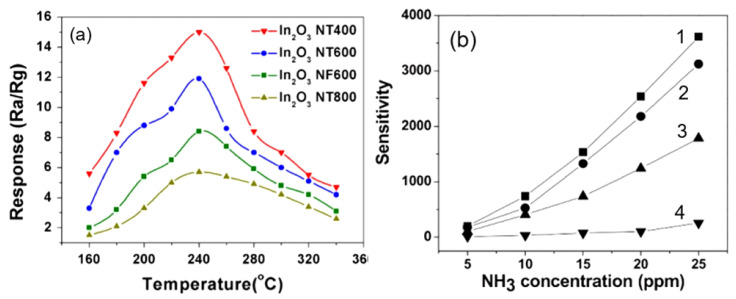
(**a**) Responses of In_2_O_3_ nanofiber-based sensors to 50 ppm formaldehyde as a function of operating temperature and the temperature of calcination (*t* = 3 h, *T_c_* = 400–800 °C). Reprinted with permission from [171]. Copyright 2016 Elsevier. (**b**) Conductivity response to NH_3_ (5–25 ppm) at room temperature for four types of gas sensors based on In_2_O_3_ nanostructures: 1—broken In_2_O_3_ hollow nanofiber; 2—regular In_2_O_3_ hollow nanofiber; 3—In_2_O_3_ nanofiber; 4—In_2_O_3_ nanoparticles. Adapted with permission from [140]. Copyrights 2007 Wiley.

The use of hollow fibers is another effective solution for improving the parameters of sensors, since this approach provides significant growth in the area of the active surface. In this case, the gas molecules can interact with the inner and outer surfaces of the nanofibers. For example, it was shown that TiO_2_ hollow fibers (HFs) exhibited a higher response to CO at room temperature compared with solid fibers. Reducing the diameter of nanofibers and the wall thickness of hollow nanofibers is also a method to improve sensor performance. This effect is due to the fact that a decrease in the diameter of NFs contributes to a decrease in both the size of crystallites formed in the fiber (increase in sensor response) and the time of gas diffusion into the fiber (decrease in response time). Really, it was established that In_2_O_3_ and ZnO NFs with a smaller diameter (≈50–100 nm) and thinner walls (≈10 nm) exhibited an enhanced conductometric response compared with larger-diameter NFs (≈500 nm) toward formaldehyde, CO and NO_2_ [173,174,175,176,177]. How important it is to provide gas access to the inner surface of hollow nanofibers was shown by experiments performed by Du et al. [140]. According to [140], broken hollow nanofibers demonstrated the highest sensitivity to NH_3_ gas, which was about 20 times higher than the sensitivity of In_2_O_3_ nanoparticles (see Figure 10b).

**Figure 11:**
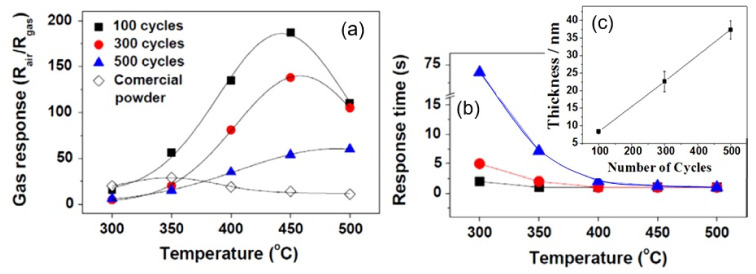
Effect of wall thickness of hollow SnO_2_ nanofibers on the (**a**) magnitude of the response to 100 ppm ethanol and (**b**) response time as a function of operation temperature; (**c**) dependence of the SnO_2_ film thickness on the number of PEALD cycles. Adapted with permission from [181]. Copyright 2010 IOP.

As shown in Figure 10b, the formation of hollow nanofibers due to an increase in the active surface area really contributes to an increase in the sensitivity of gas sensors [178,179,180]. However, it should be borne in mind that at a certain wall thickness of such fibers, it may be difficult to access the target gas at the inner surface of these hollow fibers. As a result, we encounter a situation in which we will either not observe an increase in sensitivity, or the sensor response will be too slow. The results of this effect of the wall thickness of hollow nanofibers are shown in Figure 11. Hollow SnO_2_ nanofibers were formed using SnO_2_ thin-film deposition on electrospun PAN nanofibers by plasma-enhanced atomic layer deposition (PEALD) [181]. SnO_2_ thin film-coated PAN nanofibers were annealed at 700 °C for 1 h to burn out the PAN template and crystallize the SnO_2_. It is seen that at wall thicknesses of more than 10 nm, a decrease in the sensor response and an increase in the response time to ethanol vapor are observed.

**Figure 12:**
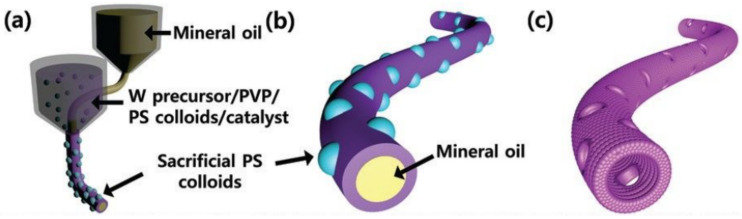
(**a**–**c**) Schematic illustrations of coaxial electrospinning using mineral oil in the core and composite solution in the shell. Reprinted with permission from [182]. Copyright 2016 Royal Society of Chemistry.

Thus, when developing hollow nanofibers intended for use in gas sensors, their wall thickness must be optimized to achieve the required parameters. The creation of pores in the walls of hollow nanofibers can also solve the problem of poor accessibility of the test gas to the inner surface of the hollow fibers. Du et al. [140] showed that pores could be created using special template deletion modes. Choi et al. [182] developed a different approach. To form macroporous hollow WO_3_ NFs, they proposed adding colloidal polystyrene (PS) particles in a solution containing a W precursor and PPV. Mineral oil was used to form the core of the nanofiber. Macropores in WO_3_ NFs were formed as a result of the removal of PS particles incorporated in the walls of hollow WO_3_ nanofibers. A schematic diagram of this process is shown in Figure 12.

Another approach to pore formation was proposed by Liang et al. [163]. They used a two-step method to form porous hollow NFs. They first synthesized hollow In_2_O_3_ NFs and then exposed them in 10% HNO_3_. As a result of this treatment, macropores appeared in the walls. Chattopadhyay et al. [183], while developing a technology for the formation of TiO_2_ nanofibers, found that the addition of a structure-directing agent (Pluronic F127—nonionic triblock co-polymer) to the electrospinning solution ultimately allows the formation of TiO_2_ nanofibers with 3D mesoporosity and a high surface area. The choice of this additive was due to the fact that this type of surfactant, having a high hydrophobic/hydrophilic ratio (>1.5), favors the formation of ordered cubic micellar aggregates in a water–ethanol mixture [184,185]. Naturally, the presence of readily accessible macro- and mesopores in nanofibers makes them more compatible for use in gas sensors.

### 3.3. Surface Modification or Surface Decoration of Metal Oxide Nanofibers

**Figure 13:**
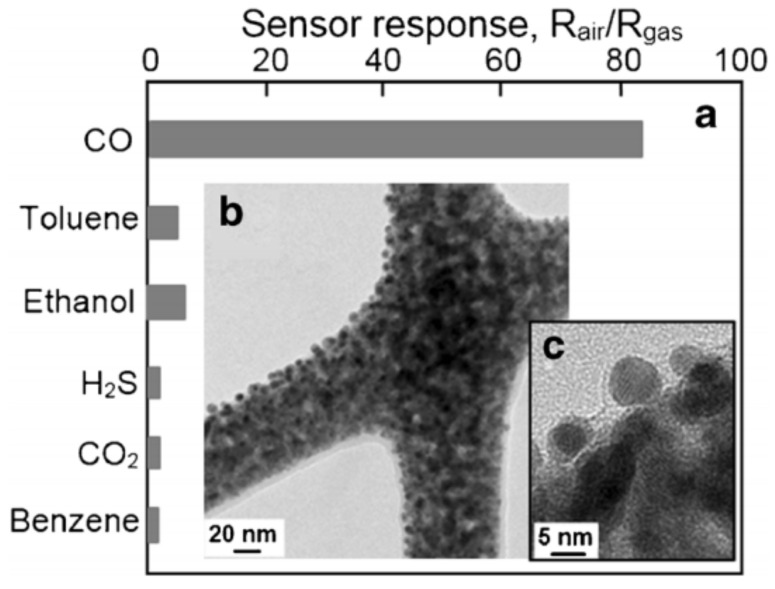
(**a**) Responses of 1.7 at% Au-loaded SnO_2_ nanofibers at *T*_oper_ = 300 °C to various reducing gases (5 ppm), and (**b**,**c**) typical TEM images of SnO_2_:Au nanofibers. Gold clusters had sizes in the range of 5–10 nm. Adapted with permission from [189]. Copyright 2014 Elsevier.

**Figure 14:**
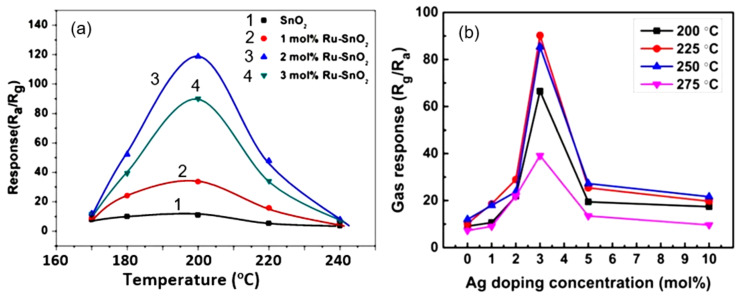
(**a**) Response of sensors based on pure and 1, 2 and 3 mol% Ru-doped SnO_2_ nanofibers to 100 ppm acetone as a function of the operating temperature. (**b**) Influence of Ag doping on the response to 5 ppm NO_2_ of electrospun WO_3_:Ag-based sensors. (**a**) Reprinted with permission from [190]. Copyright 2020: Elsevier; (**b**) Reprinted with permission from [191]. Copyright 2018 Elsevier.

Decoration of nanofibers’ surface by noble metals such as Pd, Pt, Au, Ag and Ru is also a very common method for optimizing the sensor performance of nanofiber-based gas sensors [12]. As in conventional technology [186,187,188], noble metals as effective catalysts, through a decrease in the activation energy of gas chemisorption and its catalytic oxidation, can significantly improve the selectivity (Figure 13) and sensitivity (Figure 14) of sensors to a specific gas.

As shown in Table 3, noble metals are most commonly used when developing sensors for reducing gases and vapors of organic solvents. As a rule, the maximum effect is achieved with a concentration of noble metals not exceeding 1–4 wt.% [192,193,194,195]. For example, Hu et al. [195] observed the maximum optimization effect at a Pd concentration of 3 mol% in CeO_2_ nanofibers. A concentration of 2.3 wt.% was the optimal concentration when doping In_2_O_3_ with Pt to achieve the maximum sensor response to H_2_S [196]. ZnO nanofibers doped with Ag at a concentration of 1 mol% had a maximum conductivity response to ethanol [197]. A uniform distribution of highly dispersed noble metal nanoparticles on the surface of nanofibers is also an important condition for achieving a high sensitivity and good performance of the sensors being developed [198].

#### Mechanisms of Surface Modification Influence on Gas Sensor Performances

An explanation of the observed behavior of sensors after decorating the surface with noble metals can be found in [35,186,188,199,200,201,202,203,204,205,206,207,208]. Typically, these explanations suggest the presence of two sensitization mechanisms proposed by Morrison [34] and Yamazoe [29]. They are electronic sensitization and chemical sensitization.

**Table 3:** Electrospun-based metal oxide gas sensors modified with noble metals.

Material	Dopant	Analyte gas	C, ppm	T, (°C)	Response	Detection Limit	Res./Rec. Time, s	Ref.
SnO_2_	Pd	Acetone	100	275	99	1 ppm	-	[209]
SnO_2_		Formaldehyde	100	160	19	-	2/7	[210]
In_2_O_3_		Ethanol	50	200	18	1 ppm	1/10	[130]
ZnO		CO	20	220	5.5	1 ppm	27/15	[211]
TiO_2_		NO_2_	2.1	180	38	0.16 ppm	-	[212]
WO_3_		H_2_S	1	350	1.4	1 ppm	-	[213]
WO_3_		Toluene	1	350	5.5	20 ppb	119/16	[213]
WO_3_	Pt	Acetone	2	350	4	120 ppb	-	[214]
α-Fe_2_O_3_		H_2_S	10	175	157	-		[215]
NiO		Ethanol	100	400	12	1 ppm	-	[216]
In_2_O_3_		H_2_S	600	200	1490	50 ppm	60/120	[196]
SnO_2_		H_2_S	20	300	5100	-	-	[192]
SnO_2_		Toluene	10	300	12	1 ppm	-	[217]
SnO_2_	Ag	Acetone	200	160	117	5 ppm	6/10	[218]
TiO_2_		H_2_S	1	350	120	1 ppm	-	[219]
In_2_O_3_		Formaldehyde	50	115	28	5 ppm	5/10	[220]
SnO_2_	Au	CO	10	300	19	1 ppm	-	[221]
SnO_2_		CO	5	300	84	-	22/235	[189]
In_2_O_3_		Ethanol	500	140	14	50 ppm	12/24	[222]
WO_3_		n-butanol	100	250	230	1 ppm	5–43/10–122	[223]

In chemical sensitization, the decorated noble metal acts as a promoter for the chemical interaction between the metal oxide and the analyte. The promoter increases the sensitivity to gas as it increases the rate of chemical processes, leading to a decrease in the concentration of negatively charged adsorbed oxygen [224,225]. As a rule, chemical sensitization is accompanied by direct and back-spillover effects [188,207]. Electronic sensitization is based on the existence of a potential barrier between a semiconductor and metal nanoparticles. Differences in the Fermi levels of the metal oxide and catalyst can lead to the formation of depletion/accumulation regions at the semiconductor near the metal nanoparticles. If, in the process of interaction with gas, a change in the oxidation state of the metal occurs, then this change leads to a change in the potential barrier at the metal–metal oxide interface, and therefore to a change in the conditions of current transfer in the gas-sensitive layer [224]. As a result, a sensor signal appears. It is believed that Au and Pt, when detecting reducing gases, affect sensor performance through a chemical sensitization mechanism, and Ag, Pd and Rh through an electronic sensitization mechanism. It is known that Ag, Pd and Rh have stable oxides Ag_2_O, PdO and Rh_2_O_3_ in air, which are easily reduced to metal in a reducing gas atmosphere and then easily reoxidized in an oxygen atmosphere [226]. However, the roles of sensitizers and sensitization mechanisms are not always clear. For example, Barbosa et al. [208] found that both electronic and chemical sensitization effects are relevant in Pt-decorated SnO_2_ devices. Degler et al. [207] also believe that the separation of electronic and chemical sensitization is useful for the first assessment of the role of additives, but for a complete understanding of reality, it is necessary to take into account the mutual influence of chemical and electronic properties and processes. Thus, both the electronic and chemical contributions to sensitization should be considered, rather than strictly distinguishing between electronic and chemical sensitization.

It is important to keep in mind that the activity of noble metals depends on many factors. These include parameters such as the coating thickness, cluster size, concentration of oxygen vacancies in the metal oxide support, interaction with the support, the method used to decorate the surface of metal oxides, the temperature of post-treatment and even the surface morphology and concentration of structural defects in the metal oxide itself [202,203,204,205,206,207,208]. For example, Katoch et al. [189] believe that the high sensitivity and selectivity of the response to CO of sensors based on electrospun SnO_2_ nanofibers decorated with Au were achieved due to the extremely small size of both the SnO_2_ crystallites and gold clusters, and the high density of Au nanoparticles on the surface of the SnO_2_ nanofibers (see Figure 13). It is also necessary to distinguish between the behavior of noble metals incorporated in the lattice of metal oxides and those on the surface in the form of clusters [203,207]. In particular, analyzing the characteristics of SnO_2_-based sensors doped with noble metals, such as Pt and Pd, Korotcenkov and Cho [203] concluded that the full incorporation of doping additives in the SnO_2_ lattice without forming metallic clusters on the SnO_2_ surface is the optimal condition to achieve improved performances of SnO_2_:Pt, Pt-based gas sensors.

### 3.4. Doping of Metal Oxide Nanofibers

**Figure 15:**
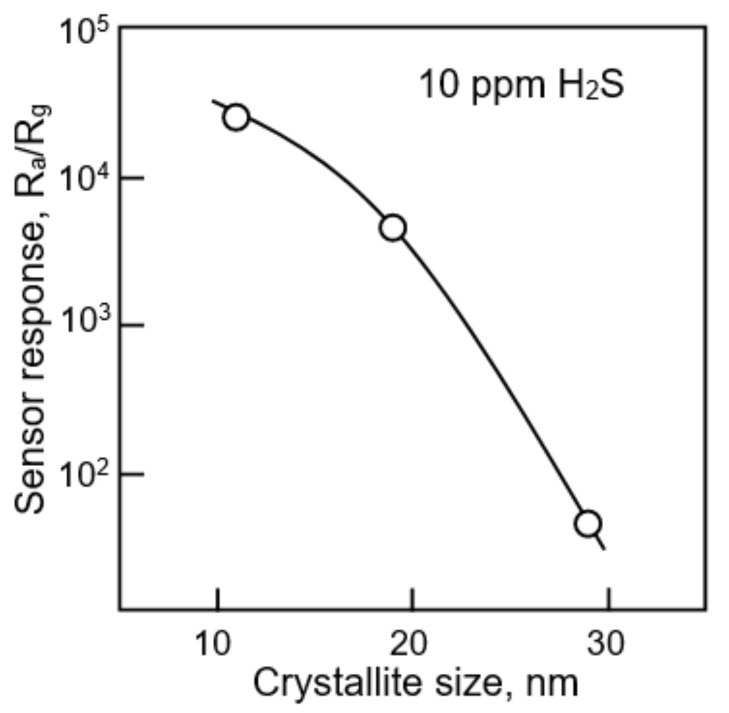
Conductometric response of CuO-SnO_2_ composite nanofibers with crystallites of different sizes to 10 ppm H_2_S gas. Adapted from [170].

As we noted above, the size of the crystallites formed in the nanofiber has a significant effect on the magnitude of the sensor response. How important this is can be judged from the results shown in Figure 15. It is seen that an increase in the crystallite size in a SnO_2_-CuO composite from 11 to 29 nm is accompanied by a decrease in the sensor response to H_2_S from 3·10^4^ to ~40, i.e., almost 10^3^ times.

In the traditional technology used for sensor fabrication, one of the methods for reducing the size of crystallites and stabilizing their size during heat treatment is doping of metal oxides [55,203]. Experiments have shown that this approach also works successfully in the manufacture of nanofiber-based sensors. For example, Zhao et al. [227], when doping α-Fe_2_O_3_ with Ca, observed a decrease in the crystallite size from 31 to 7 nm, with an increase in the Ca content in the range of 0–15 mol%. Shan et al. [228] found that Fe_2_O_3_ doping with La (5–10 wt.%) was accompanied by a decrease in the size of Fe_2_O_3_ crystallites from 14.7 to 8.6 nm. Mohanapriya et al. [229] reported that SnO_2_ doping with Ce (3–9 mol%) led, in addition to a decrease in the crystallite size and nanofiber diameter, to a significant increase in the area of the active surface (Table 4), i.e., optimization of those parameters that contribute to the growth of the sensor response. It is clear that not only the size of the crystallites is responsible for the growth of the sensor response. For example, Cheng et al. [230], investigating the effect of Fe_2_O_3_ doping with Eu in the range 0–5 wt.% on the sensor response to acetone, found that the maximum decrease in the crystallite size occurs at 5 wt.% Eu, while 3 wt.% Eu is the optimal concentration to achieve the maximum sensor response to acetone [230].

**Figure 16:**
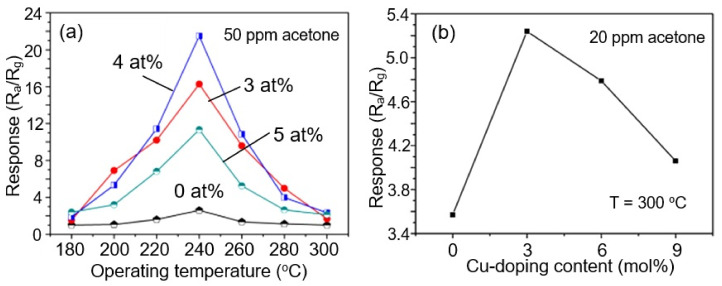
(**a**) Responses of gas sensors based on pristine and Ce-doped (3, 4 and 5 at %) α-Fe_2_O_3_ hollow nanofibers to 50 ppm acetone under different temperatures. (**b**) Response values of the pure, 3, 6 and 9 mol% Cu-doped WO_3_ hollow fibers to 20 ppm of acetone at *T*_oper_ = 300 °C. (**a**) Adapted with permission from [238]. Copyright 2014 RSC; (**b**) Adapted with permission from [239]. Copyright 2014 Elsevier.

As a result of numerous studies, it was found that doping of metal oxide nanofibers by rare-earth metals (Yb, Sr, Ce, Pr, Er, Sm, La) [12,113,122,125,229,231,232] and transition metals (Fe, Y, Ni, Cu, Co, Mn) [12,233,234,235,236,237] is indeed a powerful tool for enhancing the sensor response (see Figure 16), and, in some cases, for improving selectivity. For example, by increasing the concentration of Ni in the range 0–10 atom% in SnO_2_ NFs, Cheng et al. [234] obtained a more than 5-fold increase in the sensor response to acetone. Zhao et al. [227], due to doping of α-Fe_2_O_3_ with Ca (7 mol%), also managed to increase the response to ethanol and acetone at 200 °C by almost five times. The increase in the sensor response to other gases was significantly less.

**Figure 17:**
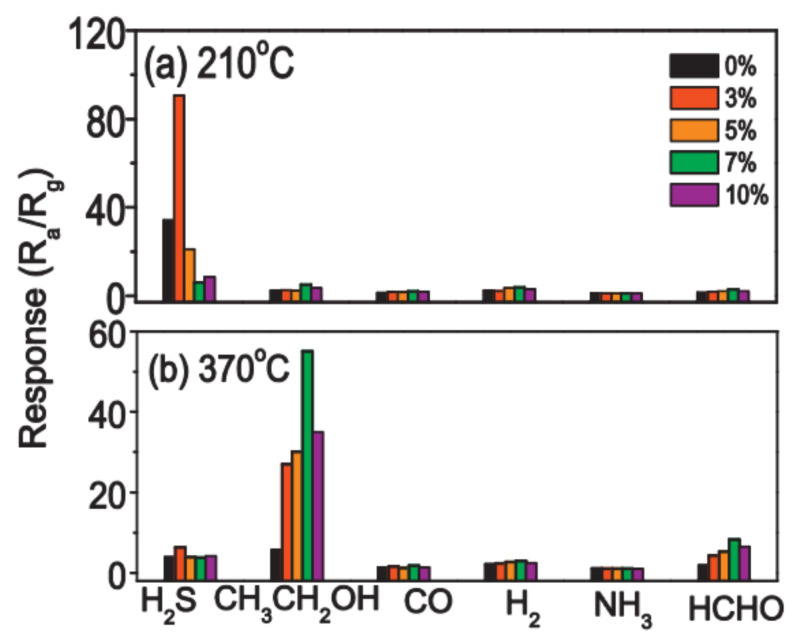
The selective test of SnO_2_:Ce nanofiber-based sensors toward (**a**) 20 ppm H_2_S and 200 ppm ethanol, CO, H_2_, NH_3_ and formaldehyde at T_oper_ = 210 °C, and (**b**) 200 ppm H_2_S, ethanol, CO, H_2_, NH_3_ and formaldehyde at *T*_oper_ = 370 °C. Reprinted with permission from [240]. Copyright 2013 Elsevier.

It is important to note here that the doping concentration providing the maximum optimization effect is not the same for all cases. It depends on the metal oxide used, the operation temperature and the gas to be detected. This effect is well demonstrated in Figure 17, which shows the effect of doping SnO_2_ with Ce on the sensor response to various gases at two temperatures [240]. It can be seen that, at low temperatures, the optimization effect of doping manifests itself in the detection of H_2_S, and the most effective is doping with Ce with a concentration of 3%, while at *T* = 370 °C, doping has a maximum effect on the sensitivity to ethanol, and this effect occurs at a doping concentration of 7%. This means that for each test gas and operating temperature, it is necessary to conduct independent research to select the optimal doping conditions.

#### Influencing Mechanism of Nanofiber Doping

Our understanding of the influencing mechanisms of the processes occurring in metal oxides during bulk doping on the effects of gas sensitivity is still insufficient for their detailed description. The influence of doping on the structural, electrophysical and gas sensing properties of metal oxides is too multifactorial. It was found that doping affects all parameters of metal oxides on which the magnitude of the sensor response depends [28,186]. Therefore, the choice of dopant and its concentration must be approached very carefully.

First, when choosing the doping element, it must be kept in mind that some additives have donor or acceptor properties that affect the concentration of charge carriers, and hence the Debye length.

Second, other additives are amphoteric impurities that have a major effect on the sensor response through changes in the crystallite size and the structure of the gas-sensitive layer.

Third, additives exhibit increased catalytic activity, while, fourth, dopants, when introduced into the metal oxide, can lead to an increase in porosity and better gas permeability.

Fifth, doping with the doping element is accompanied by the generation of structural defects that improve the adsorption properties of the surface. For instance, Cr ions act as an acceptor in TiO_2_, decreasing the electron concentration, but Nb ions act as a donor in TiO_2_, increasing the electron concentration.

It should not be forgotten that some impurities hinder the growth of crystallites during heat treatment, while others stimulate this growth, as occurs when doping TiO_2_ with Ta and Vo, respectively [55,60].

**Figure 18:**
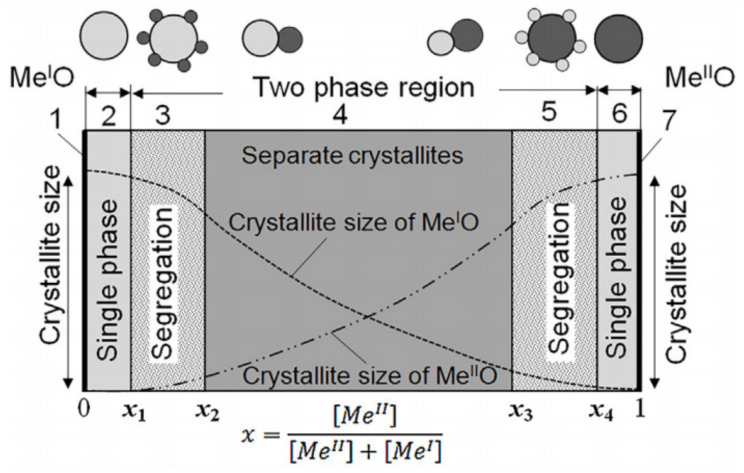
Scheme of mutual distribution of components in M^I^O−M^II^O nanocomposites. Possible transformation of the crystallite size and the grain structure of the major components of the nanocomposite is also shown in the figure. x_1_ and x_4_ correspond to the solubility limits of *Me^II^* in Me^I^O and *Me^I^* in Me^II^O. Reprinted with permission from [60]. Copyright 2017 Elsevier. Idea from Refs. [51,241].

When choosing the concentration of the dopant, one should clearly understand what structure of the metal oxide matrix is to be worked with and what consequences for the gas-sensitive material and the sensor may arise if an additional phase is introduced into the gas-sensitive material. Depending on the concentration and the type of introduced additional components, they can form doped metal oxide or solid solutions based on the main phase. They can also create a segregation of the doping element in the form of metal or metal oxide clusters on the surface of the crystallites of the main phase or form a mixture of the crystallites of two oxide phases. In the latter case, heterojunctions are formed in the gas sensing matrix. If we consider the structure of the metal oxide matrix formed by two metals, *Me^I^* and *Me^II^*, then, depending on the concentration of these metals in the composite, one can identify seven areas with radically different properties. A diagram illustrating the appearance of these areas is shown in Figure 18.

**Figure 19:**
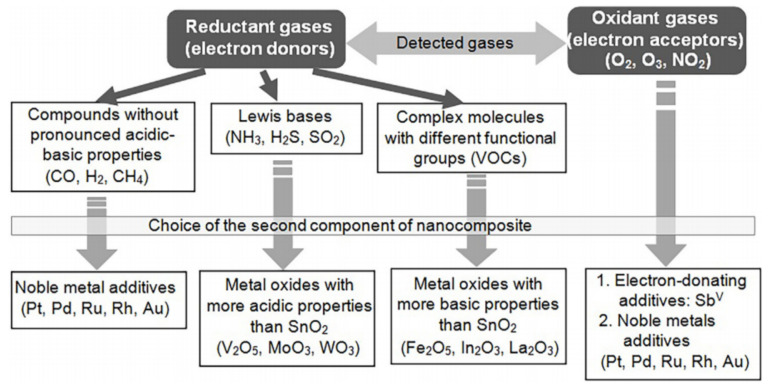
Choice of the second component of SnO_2_-based nanocomposite with allowance for the properties of the detected gas. Reprinted with permission from [60]. Copyright 2017 Elsevier. Idea and data from Ref. [51].

Unfortunately, we cannot say what dopant and what structure of the gas sensing matrix from those shown in Figure 18 are optimal for the gas-sensitive effects, since with appropriate optimization, it is possible to improve one or more sensor parameters when doped with a wide variety of additives. Undoubtedly, the criteria for the selection of doping additives proposed by Rumyantseva and Gaskov [51] can be used (see Figure 19).

Rumyantseva and Gaskov [51] believe that the interaction of a semiconducting oxide with the gas phase is described through the formation of the surface complexes, and the decisive role in this process belongs to the chemical nature of the modifier and its reactivity in acid–base or redox reactions. However, this approach does not explain all gas-sensitive effects observed when using nanocomposites based on doped metal oxides. Moreover, often the same gas sensing effect can be achieved using additives with radically different physicochemical properties. Undoubtedly, when developing gas sensors based on doped metal oxides, we can also follow the recommendations presented in Table 5 [60]. However, these recommendations are too general and do not reflect the specificity of the interaction of the composite with a specific gas. Based on the available information, it can be stated that in most cases, the maximum conductivity response of gas sensors based on the doped metal oxide is observed at a concentration of the second metal near its solubility limit in the basic oxides [60,203,242,243]. This information is presented in Table 6. Other useful information regarding the features of the effect of metal oxide doping on their gas-sensitive properties can be found in [199,200,207,244,245,246].

### 3.5. Heterostructures and Core–Shell Structures in Nanofiber-Based Gas Sensors

Using conventional technology, in a number of cases, it was possible to significantly improve the parameters of sensors due to the formation of heterostructures and the synthesis of core–shell structures [60]. The same approach has been tried with electrospinning technology [12]. As a result, various heterostructures, such as ZnO–SnO_2_, CuO–SnO_2_, CuO–TiO_2_, In_2_O_3_–CeO_2_ and Al_2_O_3_–In_2_O_3_ [247,248,249,250,251,252,253], and core–shell structures, such as ZnO–SnO_2_, SnO_2_–In_2_O_3_, ZnO–TiO_2_, Fe_2_O_3_–NiO, CuO–TiO_2_ and Co_3_O_4_–Fe_2_O_3_ [254,255,256,257,258,259,260], have been synthesized. It is important to note that such structures can be created both in the electrospinning process and using the principles of post-treatments. For example, Lu et al. [251] synthesized nanofibers of a ZnO–SnO_2_ composite by electrospinning. At the same time, Qi et al. [261] fabricated In_2_O_3_–SnO_2_ heterostructures by dipping the electrospun In_2_O_3_ NFs in the Sn(OH)_4_ sol solution. The same approach was used to prepare TiO_2_ nanofibers decorated with WO_3_ nanoparticles [262]. Subsequently, nanofibers with the specified structure were used in the manufacture of gas sensors (see Table 7).

Testing has shown that, under certain conditions, heterostructures and core–shell structures do improve gas sensor performances. For example, ZnO–SnO_2_ composite HFs, synthesized by Wan et al. [248], exhibited high sensitivity to ethanol at 260 °C, with a fast response and recovery (*τ*_res_= 4–7 s, *τ*_rec_ = 4–5 s). Additionally, most importantly, these ZnO–SnO_2_ hollow NF-based sensors showed excellent selectivity to ethanol as compared with acetone, ammonia, glacial acetic acid, DMF and formaldehyde [248]. This is an important advantage of such sensors, since one of the problems with ethanol gas sensors is their similar sensitivity to acetone.

**Table 6:** Solubility limits for metals in metal oxides most promising for gas sensor applications.

Metal Oxide	Addition	Solubility Limit	Ref.
SnO_2_	In	4–10%	[263,264]
	Mn	~5–6%	[265]
	Fe; Ni; V; Mo	<5%	[241,266,267,268,269,270]
	Nb	~3%	[271]
	Co; Cr	~0.5–3%	[272,273,274]
	Cu; Al	<1%	[243,275]
	Si	1%	[276]
In_2_O_3_	Fe	~20%	[277,278]
	Ga	10–12%	[279]
	Sn	~8%	[263,280,281]
	Nb; Mo	1–3%	[282,283,284]
	Co	~1%	[285]
	Cu	<<1%	[286,287]
ZnO	Co, Mn	13–30%	[288,289]
	Fe	2–20%	[288,290]
	V	3–15%	[289,290]
	Sn	4–8%	[291]
	Cr	~6%	[290]
	Ni, Ti	~3%	[288,290]
	Al	0.3–2.0%	[292,293]
	In	<1%	[294]
	Ga	0.5%	[293]
	Cu	<0.2%	[290]

*Source*: data extracted from [60].

**Figure 20:**
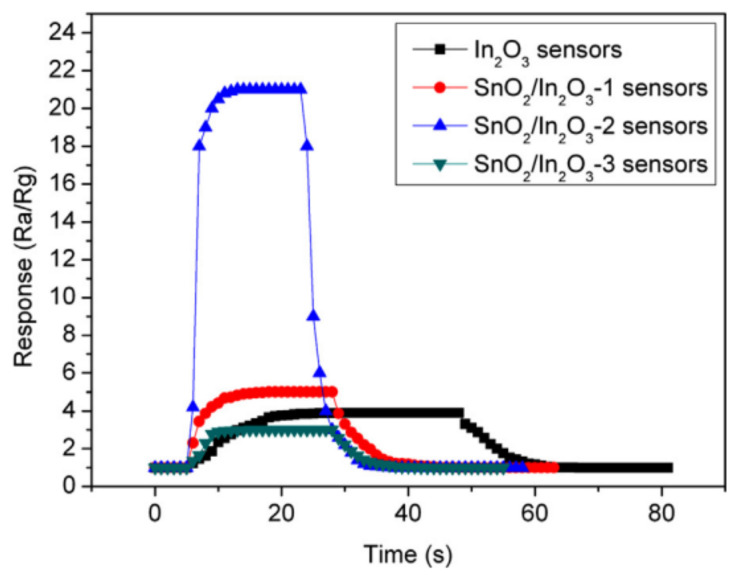
Response–time curves of In_2_O_3_ nanofiber sensors decorated with SnO_2_ to 1 ppm NH_3_ at room temperature: SnO_2_/In_2_O_3_—1–7.5 at.% Sn; SnO_2_/In_2_O_3_—2–16 at.% Sn; and SnO_2_/In_2_O_3_—3–21 at.% Sn. Reprinted with permission from [261]. Copyright 2014 Elsevier.

Feng et al. [252] showed that In_2_O_3_–WO_3_ heterojunction NFs had an increased sensitivity to acetone compared to pure WO_3_ NFs. In_2_O_3_–SnO_2_ heterojunction NFs were highly sensitive to NH_3_ [261] and CO [253]. It is only important to know that an improvement in gas sensor performances is observed only under optimal conditions for the formation of heterostructures. For example, as seen in Figure 20, an increase in the sensor response of In_2_O_3_–SnO_2_ heterostructures to NH_3_ was observed only under the condition that the SnO_2_ concentration in the heterostructure was 16 at.%.

Similarly, CuO–SnO_2_ NFs exhibited a very high response toward H_2_S compared with pure SnO_2_ NFs [249]. As with conventional metal oxide-based sensors, the high sensitivity to H_2_S of CuO-based heterostructures, such as CuO–SnO_2_ or CuO–In_2_O_3_, is connected with the phase transformation of CuO, a p-type semiconductor, in CuS with metallic characteristics when interacting with H_2_S gas. The result is a significant change in the device structure, from a p–n heterostructure to a metal–semiconductor configuration. The opposite process takes place in an oxygen atmosphere.

**Figure 21:**
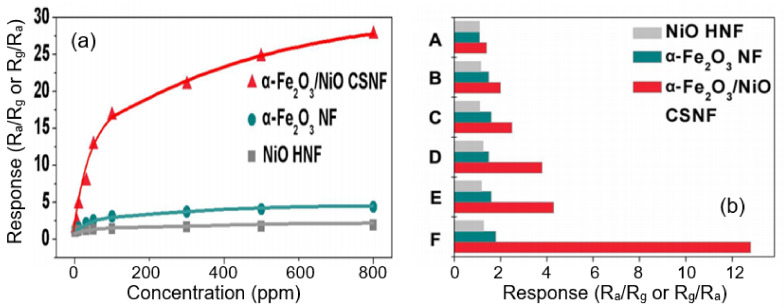
(**a**) Responses of the sensors based on NiO HNF, α-Fe_2_O_3_ NF and α-Fe_2_O_3_/NiO CSNF to HCHO gas (*T*_oper_ = 240 °C); (**b**) the selectivity of α-Fe_2_O_3_/NiO CSNF, α-Fe_2_O_3_ NF and NiO HNF to 50 ppm different gases ((A) ethyne, (B) ammonia, (C) trichloromethane, (D) methylbenzene, (E) ethanol, (F) formaldehyde). Reprinted with permission from [255]. Copyright 2015 RSC.

Core–shell α-Fe_2_O_3_/NiO nanofibers synthesized by Cao et al. [255], core–shell CuO/TiO_2_ nanofibers synthesized by Deng et al. [250] and core–shell In_2_O_3_/SnO_2_ nanofibers synthesized by Wan et al. [259] exhibited significantly improved conductivity responses to formaldehyde and selectivity performances in comparison with NiO hollow nanofibers, and α-Fe_2_O_3_, CuO, TiO_2_, In_2_O_3_ and SnO_2_ nanofibers. For example, at an operation temperature of 240 °C, Fe_2_O_3_–NiO-based sensors had fast response–recovery behavior (~2 s and ~9 s) (see Figure 21). Sensors based on α-Fe_2_O_3_/TiO_2_ core–shell structures had a higher response and better selectivity to a low concentration of TMA at 250 °C in comparison with pristine α-Fe_2_O_3_ and TiO_2_ sensors [260].

**Table 7:** Some sensing properties of composite and heterostructure-based electrospun metal oxide NFs.

Material	Gas	Conc. (ppm)	T, (°C)	Response (Ra/Rg)	Ref.
TiO_2_–ZnO	O_2_	10,000	300	20	[295]
ZnO–rGO	NO_2_	5	400	119	[79]
p-In_2_O_3_–TiO_2_	NO_x_	97	25	40	[296]
Al_2_O_3_–In_2_O_3_		97	25	100	[254]
CuO–In_2_O_3_	H_2_S	5	RT	9170	[297]
CuO-SnO_2_		10	300	25799	[170]
ZnO–CuO		10	150	4490	[298]
SnO_2_–CeO_2_		20	210	90	[240]
CuO–ZnO	CO	0.1	300	7	[299]
SnO_2_–RGO		1	200	10	[300]
TiO_2_–ZnO		0.1	375	15	[301]
SnO_2_–ZnO		10	350	11	[302]
SnO_2_–MWCNT		50	25	1.3	[303]
p-NiO–n-SnO_2_	H_2_	100	320	13	[304]
In_2-x_Ni_x_O_3_	C_2_H_5_OH	100	180	80	[305]
Cr_2_O_3_–ZnO		100	300	24	[116]
In_2_O_3_–ZnO		100	210	25	[306]
ZnO–In_2_O_3_–ZnO		100	210	17	[306]
Sn-SnO_2_–Carbon		500	240	30	[307]
ZnO-TiO_2_		500	320	51	[258]
SnO_2_–ZnO	CH_3_OH	10	350	8.5	[308]
In_2_O_3_–WO_3_	C_3_H_6_O	0.8	350	1.8	[252]
SnO_2_–α-Fe_2_O_3_		100	340	31	[309]
In_2_O_3_–WO_3_		0.4	275	1.3	[252]
α-Fe_2_O_3_–NiO	CH_2_O	50	240	13	[255]
NiO–SnO_2_		10	200	6.3	[310]
SnO_2_–In_2_O_3_		0.5	375	2.2	[311]
SnO_2_–In_2_O_3_		50	300	115	[257]
PPy–WO_3_	NH_3_	20	100	26	[312]
SnO_2_–In_2_O_3_		1	25	21	[261]
PANI–TiO_2_		0.025	25	0.4	[313]
p-La_0_._67_Sr_0_._33_MnO_3_–n-CeO_2_	C_3_H_8_	20	800	75	[238]
NiO-SnO_2_	C_7_H_8_	50	330	11	[314]
La_0_._7_Sr_0_._3_FeO_3_–In_2_O_3_-SnO_2_	C_3_H_9_N	1	80	8	[315]
p-NiO–n-ZnO		100	260	892	[316]
ZnO–In_2_O_3_		5	375	119	[317]

C_3_H_6_O—acetone; C_2_H_5_OH—ethanol; CH_2_O—formaldehyde; C_3_H_8_—propane; CH_3_OH—methanol; C_7_H_8_—toluene; C_3_H_9_N—trimethylamine; PPy—polypyrrole; PANI—polyaniline; rGO—reduced graphene oxide.

In accordance with the conclusions made in [65,318,319,320,321,322,323], the main reasons for improving the parameters of sensors based on heterostructures and core–shell structures are the presence of an interface between two dissimilar materials and the combination of these heterojunctions into a network. The creation of a close electrical contact at the interface between these two components, which facilitates the equilibration of Fermi levels at the interface, usually leads to charge transfer and further expansion of the charge depletion region in contacting crystallites [318,324]. Another important factor to consider for heterostructures is synergistic behavior [325,326]. When two different components in a material are in contact with the gas phase and each exhibits its own specificity when interacting with a gas, then a situation may arise where the synergistic effect of the two-component system can be greater than the effect observed in these elements separately. These two factors are the basis of unique effects that can lead to an improved performance of gas sensors based on heterostructure-based materials.

However, prediction and analysis of the gas-sensitive characteristics of sensors based on heterostructures are difficult tasks, since, depending on the properties of the contacting materials, their ratio and methods for preparing heterostructures, radically different scenarios can be realized. In the case of mixed metal oxides, either of the two metal oxides can predominate in conductivity if they offer a conduction path that minimizes electrical resistance. It is also possible that conduction occurs through both metal oxides, and thus the charge transfer must cross heterojunctions formed at grain boundaries between the two materials [58,318].

In the case of doped and loaded metal oxides, conduction occurs exclusively through the supporting metal oxide, and the sensitization effect arises from the chemical and electrical interaction between the supporting metal oxide and the additive that form the heterostructure [207]. In addition, depending on the properties of the contacting materials, both *n*–*n* and *p*–*n* heterojunctions can be formed, with their own specific interaction with the test gas.

Processing routes also have a strong influence on the electrophysical and gas sensing characteristics. As it was indicated above, heterostructures can be prepared directly during electrospinning or by loading a second metal oxide on the surface of an already electrospun metal oxide. Although two materials in these two formed heterostructures can have the same nominal composition, the behavior of these heterostructures can be radically different [60]. In core–shell structures, the influence of the thickness and gas permeability of shell layers is also added. Therefore, despite the large variety of heterojunction-based sensors formed and tested, there is still no clarity in the understanding of the complicated gas sensing mechanisms in such devices. As a result, it is difficult to predict the gas-sensitive effect that may occur during the formation of heterostructures and core–shell structures.

### 3.6. Post-Treatments of Nanofibers

It is important that, in addition to influencing the structure and composition of nanofibers, various post-modification methods can be used to control gas-sensitive properties. For instance, Du et al. [327] proposed exposing the formed In_2_O_3_ nanofibers to RF low-temperature oxygen plasma (f = 13.6 MHz, P = 450 W, t = 30 min). They found that this treatment of nanofibers was accompanied by an increase in the sensor response to acetone. Treatment in oxygen plasma, leading to an increase in the concentration of chemisorbed oxygen on the surface of metal oxides, should indeed promote an increase in the sensor response to reducing gases. However, this state of the surface cannot be stable, especially when interacting with reducing gases. At the same time, Du et al. [327] claimed that the sensors have stable parameters. This means that parameter optimization is of a different nature. Really, Du et al. [327] found that treatment in oxygen plasma has a significant effect on the structure of nanofibers, which manifests itself in a decrease in the size of In_2_O_3_ crystallites with an increase in the diameter of nanofibers, leading to an increase in their porosity (Table 8). Apparently, exactly these changes are responsible for the observed increase in the sensor response. Du et al. [327] explained this effect by etching the surface of In_2_O_3_ crystallites with oxygen plasma. However, it is not clear how etching can be accompanied by an increase in the diameter of nanofibers.

**Figure 22:**
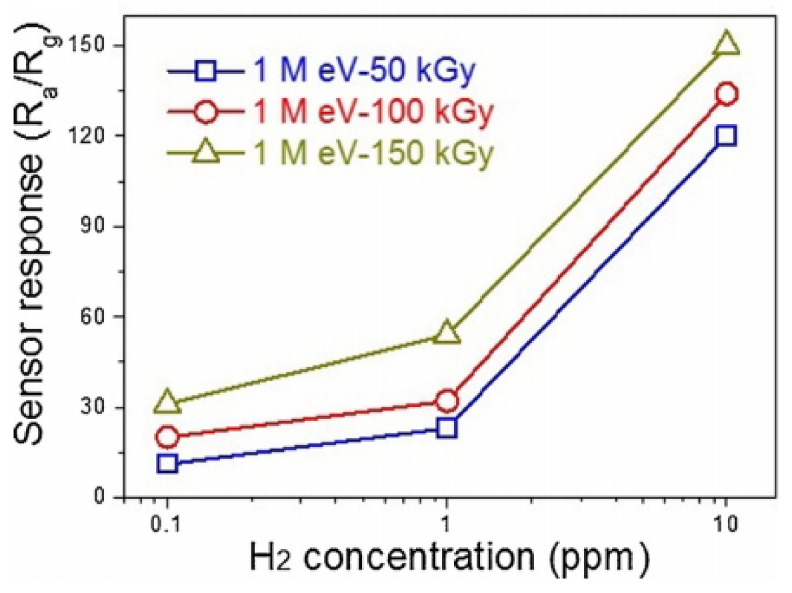
Response of ZnO NF sensors irradiated at different e-beam doses to 0.1, 1 and 10 ppm H_2_ at *T*_oper_ = 350 °C. Reprinted with permission from [328]. Copyright 2019 Elsevier.

Kim et al. [328] believe that high-energy electron-beam irradiation (E = 1 MEv, 50–150 kGy) of prepared metal oxide nanofibers can also be used to improve the sensor performance of nanofiber-based devices. They established that the response of a sensor based on ZnO nanofibers to 10 ppm H_2_ increased after electron-beam irradiation at a dose of 100–150 kGy. However, it must be admitted that the increase in the sensor response was insignificant (see Figure 22). Kim et al. [328] assumed that this improvement took place due to surface and structural defects generated by e-beam irradiation. This manifested itself in an increase in the surface areas of the samples and a decrease in the sensor resistance after electron-beam irradiation.

**Figure 23:**
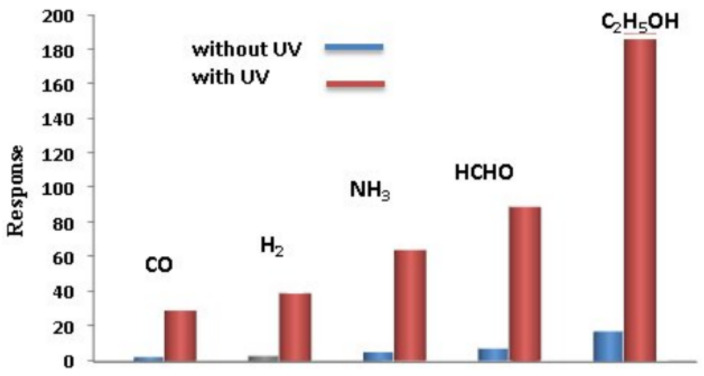
The response of TiO_2_ nanofiber sensor under dark and UV illumination conditions to several gases with concentration of 75 ppm. Reprinted with permission from [141]. Copyright 2015 Elsevier.

**Figure 24:**
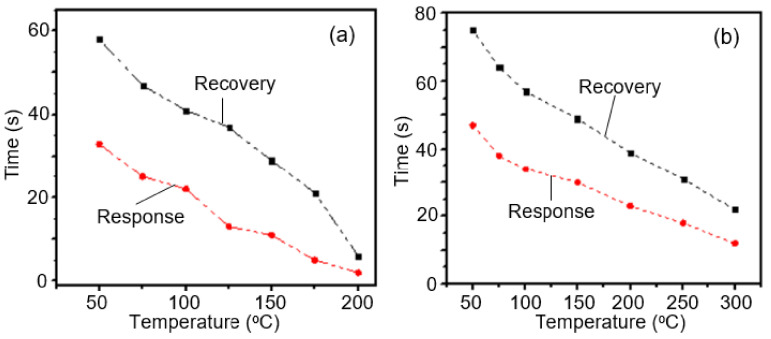
The response and recovery times of TiO_2_ nanofiber sensor in 50 ppm hydrogen gas as a function of temperature (**a**) with UV and (**b**) without UV irradiation. Reprinted with permission from [141]. Copyright 2015 Elsevier.

Nikfarjam and Salehifar [141] found that UV irradiation of sensors during gas detection, as in the case of conventional metal oxide gas sensors, has a significant optimization effect (see Figure 23). For example, by UV irradiation (λ = 390–410 nm), the response of a TiO_2_ nanofiber-based sensor to H_2_ was increased 10-fold, and response and recovery times were reduced by three–six times (Figure 24). For TiO_2_ sensors modified with Au, the optimization effect was even greater. For CO, the response of Au/TiO_2_ sensors under the influence of UV irradiation increased by about 20 times compared to dark conditions. In addition, the operating temperature was reduced from about 290 to 170 °C. The optimization effect of UV irradiation was also observed in the detection of formaldehyde by SnO_2_/TiO_2_ [329] and SnO_2_/ZnO heterostructure-based sensors [330].

The mechanism of the UV irradiation influence on sensor characteristics is described in sufficient detail in [187,331,332,333,334,335,336]. In [52,187,337,338], it was suggested that the absorbed photons modulate the receptor function of the MOX by (1) excitation of the solid (in other words, enhancement of the concentration of charge carriers in the solid), (2) the formation of highly reactive surface radicals, (3) a change in both the surface density of adsorption sites of various types and the surface coverage by the gas-adsorbed species and (4) the filling of defects with charge carriers. For example, UV photoactivation can promote oxygen desorption and increase the population of target gas molecules on the metal oxide surface [339].

It is important to note that light activation is most effective at low operating temperatures of sensors, and when the concentration of free electrons in metal oxide nanostructures is low, active oxygen species are formed with difficulty, and the dynamics of gas desorption is rather slow. That is why metal oxide gas sensors operated in the dark generally exhibited poor sensitivity and long response/recovery times at room temperature. According to Comini et al. [338], photoexcitation processes can also modulate the charge transport across the grain boundaries by: (i) increasing the concentration of free charge carriers throughout the material due to the electrons remaining in the conduction band; (ii) decreasing the barrier height at the grain contacts due to the variation in the interface charge; or (iii) by increasing the probability of charge carrier tunneling through the inter-grain barriers by decreasing the depletion layer widths in the adjacent grains. However, the consistent mechanisms responsible for the photo-assisted gas sensing properties of metal oxides still need to be thoroughly studied.

Wang et al. [335] showed that a decrease in crystallite size, an increase in material porosity and the formation of heterointerfaces enhance the effect of UV irradiation on the sensor response to test gases. Considering the characteristics of metal oxide nanofibers, it can be concluded that UV photoactivation can indeed be an effective method of improving the performances of nanofiber-based sensors operated at room temperature, increasing the sensor signal and decreasing the time constants of the sensor response.

### 3.7. Stability of Nanofiber-Based Gas and Humidity Sensors

**Figure 25:**
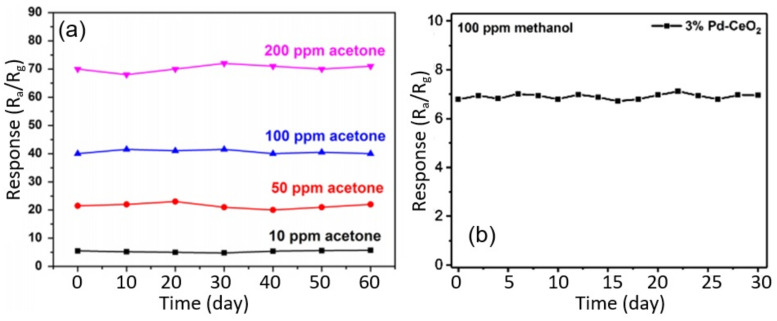
(**a**) Long-term stability of gas sensor based on 4 at.% Ce-doped α-Fe_2_O_3_ nanotubes to 10, 50, 100 and 200 ppm acetone at 240 °C. (**b**) Stability test of the 3% Pd-CeO_2_ nanofiber-based sensor to 100 ppm methanol at 200 °C. (**a**) Reprinted with permission from [340]. Copyright 2014 Elsevier; (**b**) Reprinted with permission from [195]. Copyright 2020 Elsevier.

As for the stability of the nanofiber-based sensors being developed, the studies carried out in this area have shown that gas sensors within the tested time, 1–6 months, have an acceptable stability of sensor parameters. As it is seen in Figure 25, during the test, the sensors exhibit almost constant sensor signals. This is quite understandable, since nanofiber-based sensors, by their nature, do not differ in any way from metal oxide gas sensors that have been on the market for a long time and have confirmed their high stability.

**Figure 26:**
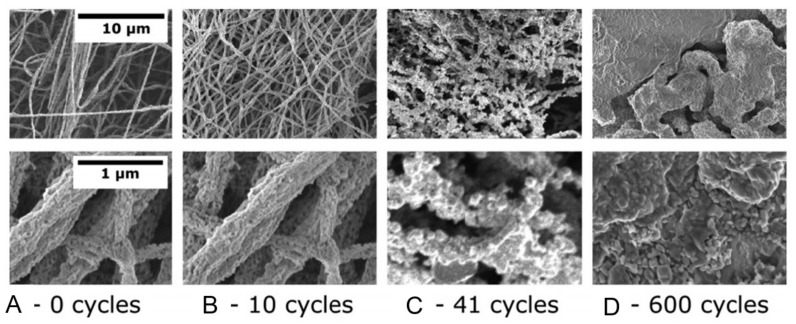
SEM images of CuO nanofibers after different numbers of H_2_S (10 ppm) sensing cycles (A → D). As-prepared CuO nanofibers had a diameter of 600–700 nm. Reprinted with permission from [341]. Copyright 2019 De Gruyter.

However, in sensors in which chemical reactions occur during the detection process, changes in the morphology of the gas-sensitive layer (nanofiber mat) and the sensor response during the operation of the sensors are possible. Seitz et al. [341], investigating the behavior of CuO nanofiber-based H_2_S sensors, found that due to phase transformations in the interaction of CuO with H_2_S (CuO ↔ CuS), these sensors undergo a dramatic morphological change during their life time (see Figure 26, A → D). Due to the high mobility of ions, fibers tend to break apart and grow to bigger agglomerates with a small area of the active surface. Interestingly, percolation-induced sensing still works for these structures but results in signals with a reduced signal-to-noise ratio.

Seitz et al. [341] believe that improving the stability of the morphology of CuO-based H_2_S sensors, and hence the stability of their parameters, is possible through generating composite fibers with other oxides which do not undergo a chemical reaction with H_2_S. This approach was used in the development of H_2_S sensors based on In_2_O_3_ [297], SnO_2_ [131,170] and ZnO nanofibers [298] modified with CuO. Unlike CuO, these metal oxides do not chemically react with H_2_S and are stable in a H_2_S atmosphere. For example, Katoch et al. [299] reported that SnO_2_–CuO-based H_2_S sensors were stable for more than six months and showed slight deviations in the parameters of various fabricated samples.

**Figure 27:**
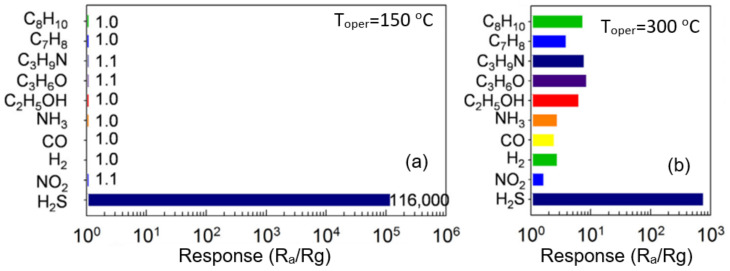
Gas responses of CuO-loaded In_2_O_3_ nanofiber sensors toward various gases at (**a**) 150 °C and (**b**) 300 °C. The response corresponds to R_a_/R_g_ ratio for reducing gases and to R_g_/R_a_ for NO_2_. Reprinted with permission from [297]. Copyright 2015 Elsevier.

Various methods can be used to modify the surface of metal oxides with CuO clusters. For instance, Katoch et al. [170] synthesized CuO–SnO_2_ composite nanofibers directly in the electrospinning process. For these purposes, they used a solution containing dehydrate (SnCl_2_·2H_2_O), copper chloride dihydrate (CuCl_2_·2H_2_O), DMF and ethanol as solvents, and acetate (PVAc) as a polymer. Yang et al. [131], in order to obtain CuO-modified hollow SnO_2_ nanofibers, immersed SnO_2_ hollow nanofibers into a Cu(NO_3_)·3H_2_O aqueous solution for 6 h at 95 °C. After that, SnO_2_ nanofibers, modified with CuO, were washed with deionized water and ethanol and dried at 70 °C for 24 h. As in CuO-based sensors, the conductometric response is determined by the reaction of the interaction between CuO and H_2_S, accompanied by a transformation from *p*-CuO to metallic CuS. However, unlike CuO-based sensors, these changes occur only in CuO clusters. After exposure to interference gases such as CO, NO_2_, acetone and alcohol, the reaction of the transformation from p-CuO to metallic CuS does not occur [131]. That is why these sensors have such a high selectivity for detecting H_2_S. For example, for sensors developed by Liang et al. [297], the ratio S_H2S_/S_gas_ reaches ~10^5^ at *T*_oper_ = 150 °C and ~10^2^ at *T*_oper_ = 300 °C (see Figure 27).

As it can be seen, the selectivity is much higher at 150 °C. However, for such low temperatures, the sensor resistance did not recover to its original value even after exposure to an air atmosphere for 100 s. The same situation was observed by Kapse et al. [342], who also reported the sluggish or incomplete recovery from H_2_S sensing at *T* < 200 °C for In_2_O_3_–CuO-based sensors. It is believed that the difficult desorption of SO_2_ is the cause of this phenomenon. This problem, as Liang et al. [297] suggested, can be solved by refreshing the sensor surface via pulse heating to 500 °C. Using this approach, Liang et al. [297] succeeded in achieving fully reversible gas sensing characteristics and in shortening the recovery time to <140 s.

## 4. Limitations of Electrospinning for Gas Sensor Design and Approaches to Resolving These Problems

There is no doubt that electrospinning is a powerful method for producing a variety of nanostructured materials and highly sensitive gas sensors. However, despite the successes achieved, essential studies are still required in this area, and many challenges have to be faced. This is due to the fact that in addition to all the advantages listed above, the electrospinning process has some limitations [343,344].

The following disadvantages of electrospinning technology are most commonly indicated:

- **First**, according to Mondal and Sharma [344], some of the disadvantages of electrospinning technology are (a) the need to use a templating carrier polymer, since direct electrospinning is not possible for all metal oxides, and (b) the limited number of polymers that can be used for the production of metal oxide nanofibers by electrospinning.

However, it must be admitted that the above cannot be attributed to disadvantages, since (a) the use of a carrier polymer makes it possible to reduce the diameter of the formed nanofibers, which means reducing the size of crystallites and thereby improving the parameters of gas sensors; and (b) the electrophysical properties of polymers do not have any effect on the properties of metal oxide fibers, since the polymers are removed after electrospinning. It is also important that the removal of polymers is well combined with the temperature conditions of the processing used in the calcination of deposited metal oxide nanofibers.

- **Second**, the variety of applications and performances of electrospun metal oxide nanofibers is limited due to their brittleness after calcination [345]. In particular, electrospun metal oxide nanofibers after calcination cannot be used in the development of sensors based on flexible substrates. However, conventional metal oxide conductometric gas sensors have the same limitation.
- **Third**, nanofibers have poor adhesion to the substrate. Electrospun fibers are also characterized by the poor interfacial adhesion properties between the nanofibers. It is known that the mat of electrospun nanofibers consists of fibers with a weak inter-fiber interaction. As a result, such a network of nanofibers has reduced mechanical properties and a high contact resistance.

**Figure 28:**
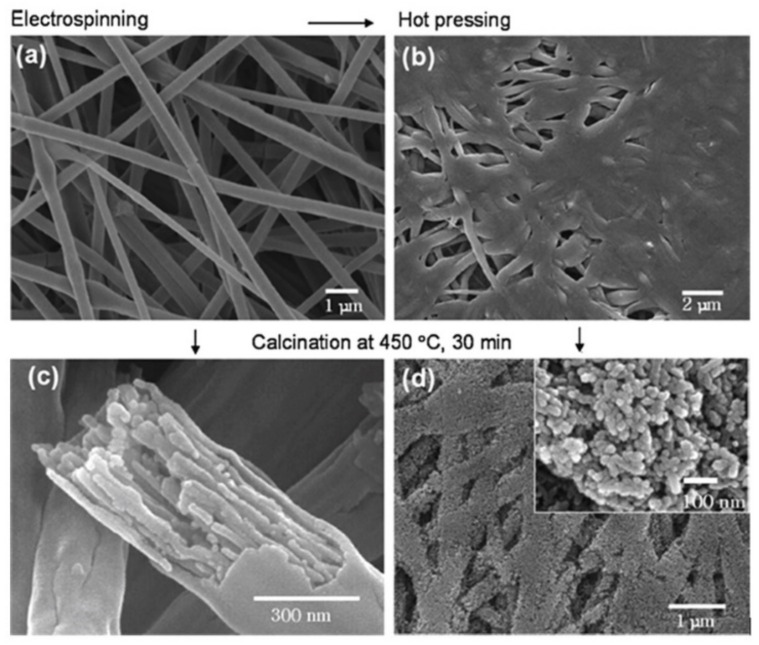
Electrospinning and hot pressing of metal oxide materials: (**a**) SEM image of as-spun TiO_2_/PVAc composite fibers fabricated by electrospinning from a DMF solution; (**b**) SEM image of TiO_2_ PVAc composite fibers after hot pressing at 120 °C for 10 min; (**c**) SEM image of unpressed TiO_2_ nanofibers after calcination at 450 °C; (**d**) SEM images with different magnifications of hot-pressed TiO_2_ nanofibers after calcination at 450 °C. Reprinted with permission from [348]. Copyright 2006 American Chemical Society.

Experiments have shown that an additional hot pressing step after the polymer–metal oxide fiber has been deposited, but before calcination, makes it possible to solve the problem of poor adhesion between the fibers in the mat, as well as between the mat and the substrate [142,143,346]. Hot pressing also helped to reduce the resistance of nanofiber–metal electrode contacts [142,143]. This was due to an increase in the contact area. However, besides improving adhesion, this treatment had an effect on the microstructure of the fibers, as shown in Figure 28 for TiO_2_-based fibers. By introducing the hot pressing step prior to calcination, an interconnected morphology of the TiO_2_/polymer composite fibers was obtained, as illustrated in Figure 28b, due to the partial melting of the polymer vehicle. Subsequent calcination resulted in the structures shown in Figure 28d. As it can be seen, strong hot pressing makes the structure of the gas-sensitive layer denser in comparison with the original structure, bringing it closer in its properties to the structure of films formed by conventional thick-film technology.

The photochemical activation proposed by Meng et al. [347] also contributed to the improvement in adhesion of metal oxide nanofibers. Using this approach, Meng et al. [347] manufactured high-performance field-effect transistors based on In_2_O_3_ nanofibers. The UV treatment enabled the stable adhesion of the nanofiber network and the formation of a clean interface. Due to their improved adhesion properties, field-effect transistors have demonstrated improved device uniformity and efficient modulation of electrical characteristics. Apparently, this approach can also be used in the manufacture of gas sensors.

According to Seitz et al. [341], improved adhesion of the fibers to the substrate is also obtained by pretreating the substrate surface in PVP solution in EtOH. They discovered this effect when forming a CuO nanofiber mat using electrospinning of the solution, containing 7 wt.% PAN and 10 wt.% Cu-2-ethylhexanoate.

**Figure 29:**
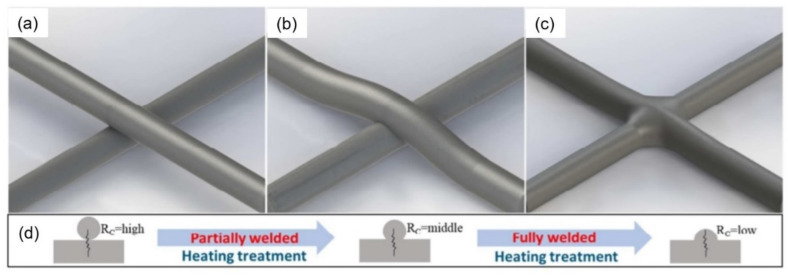
(**a**–**c**) Schematic illustration of welding process for nanofiber networks. (**d**) Contact resistance variation at the nanojoint. Adapted with permission from [349]. Copyright 2018 Royal Society of Chemistry.

Cui et al. [349] showed that a simple and efficient nanoscale welding technology can also be used to assemble metal oxide nanofibers into a large area jointed NF network with improved inter-fiber bonds and strong interfacial adhesion properties (Figure 29). They believe that the in situ crosslinking process has several advantages over the conventional solvent vapor welding or hot pressing process. This process is more efficient, much simpler and does not require expensive reagents or equipment. Cui et al. [349] proposed using an amine-hardened epoxy resin as an adhesion agent for fabrication of an In_2_O_3_ nanofiber-based network. According to Cui et al. [349], the crosslinking welding process is controlled by the spontaneous chemical reaction between polymer chains, rather than high-energy radiation. Therefore, its application is not restricted to the limited area and flatness of the substrate. It is important to note that this technology can be effective in the development of gas sensors, since it better preserves the nanofiber mat structure, which is optimal for gas sensor applications.

- **Fourth**, to date, it remains a challenge to produce nanofibers with diameters smaller than 20–50 nm by the existing electrospinning technique.

**Figure 30:**
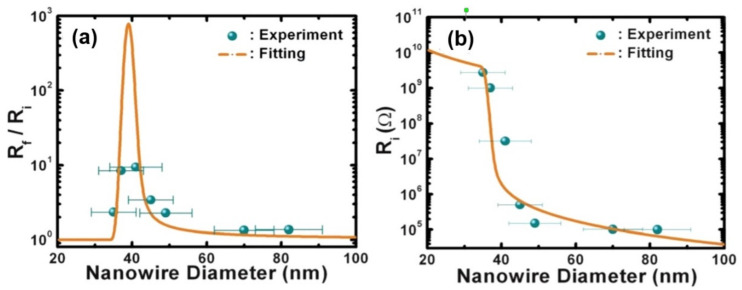
Influence of fiber diameter (**a**) on the response of WO_3_ NF-based sensor to 150 ppb NO gas, and (**b**) on the initial resistances of the sensors. Reprinted with permission from [27]. Copyright 2012 RSC.

It has been found that both the response time and sensitivity improve significantly with a decreasing nanofiber diameter [237]. This effect occurs due to the fact that the diameter of the nanofibers affects the size of the crystallites formed in the nanofiber during calcination. The smaller the diameter, the smaller the crystallite size. As it is known, the size of crystallites is one of the main factors that control the response of conductometric gas sensors [28,36,55]. In addition, a smaller diameter results in a faster response due to the faster diffusion of gas molecules through the nanofiber. However, an electrospun fiber of the conventional process typically has a diameter in the range of 100–500 nm. Therefore, numerous studies focused on the development of a reliable method for producing nanofibers with an extremely small diameter in large quantities and with a uniform size. However, it is quite possible that such thin nanofibers (<20 nm) will not be needed. For example, Vuong et al. [27], investigating the gas-sensitive characteristics of WO_3_ nanofibers, found that the maximum sensor response to NO at 300 °C was observed with a nanofiber diameter of ~40 nm (see Figure 30a). A sharp increase in the resistance of nanofibers corresponded to this diameter (Figure 30b).

As a rule, the maximum sensor response correlates well with the ratio of the crystallite size and the Debye screening length [30,32,54]. This means that with a corresponding influence on the concentration of charge carriers in crystallites through doping of metal oxides, the maximum sensor response can be achieved even with a larger crystallite size, and therefore with a larger diameter of nanofibers. For example, Zhang et al. [350] showed that doping with Mg can be used to reduce the carrier concentration in In_2_O_3_ crystallites formed by electrospun nanofibers.

- **Fifth**, electrospinning does not provide highly reproducible nanofiber parameters such as the nanofiber diameter and the response of sensors based on these nanofibers [25,351].

As it was shown earlier, too many factors affect the parameters of the formed metal oxide nanofibers [352,353]. This means that the control of all technological parameters is a necessary step in the manufacture of a gas sensor to achieve an acceptable reproducibility of the sensor characteristics. Much has been conducted in recent years to improve the reproducibility of nanofiber parameters. For example, Demir et al. [354] proposed increasing the temperature of the solution. They found that the diameters of the fibers obtained from the polymer solution at high (70 °C) temperature were much more uniform than the diameters obtained at room temperature. However, a solution to the problem of the low reproducibility of nanofiber parameters that satisfies all the requirements has not yet been found.

- **Sixth***,* the complexity of fabrication also creates certain difficulties in using electrospinning technology to form gas-sensitive layers on the sensor platform. This also includes the impossibility of forming gas-sensitive layers localized in certain places of the platforms of gas sensors by this method.

**Figure 31:**
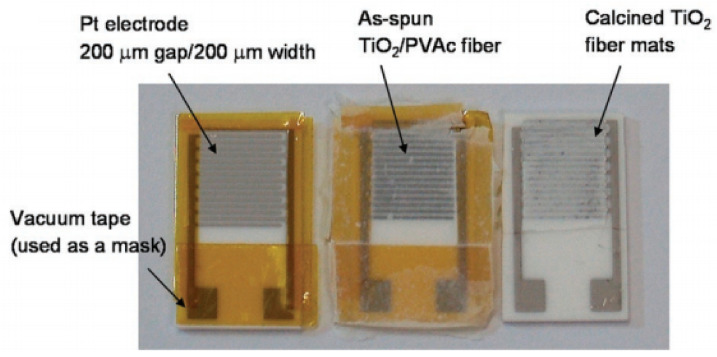
Optical micrographs of gas sensor test devices (10 × 15 mm) with TiO_2_ nanofiber mats after different processing steps. Reprinted with permission from [348]. Copyright 2006 ACS.

As it follows from the description of electrospinning technology, it is really difficult to spin the fibers in a small sensing area of the microsensor. This method is indeed more suited for the formation of a nanofiber mat on areas that significantly exceed the traditional dimensions of gas sensors, especially those manufactured in microelectronic design. For example, Kim et al. [348] fabricated TiO_2_ nanofiber-based gas sensors using a platform similar in appearance to that often used in the development of conventional thick-film gas sensors (see Figure 31). However, its dimensions of 10 × 15 mm are much larger than the platforms used in conventional gas sensors. The other result could not be expected, since with a standard distance between the electrode and the collector of 10–15 cm, the deposition area cannot be less than 10 cm^2^.

Zhang et al. [105], analyzing the reasons for these limitations, came to the conclusion that this problem can be solved only by reducing the distance between the spinneret and the collector. It was found that by reducing the distance between the collector and spinneret to 500 μm–5 cm, so-called near-field electrospinning can be realized [355,356,357]. This process, due to the absence of bending instability, allows the application of fibers with a high spatial resolution, by matching the average motion speed (S_J_) of the jet with the relative speed (S_R_) between the collector and the spinneret. Compared with far-field electrospinning, near-field electrospinning offers a number of advantages [352]. They are as follows:

(i) Substantial reduction in the applied voltage;
(ii) The ability to accurately position the fibers over a relatively large area with minimal material consumption;
(iii) The ability to manipulate the spatial positions of the fibers along all three directions, X, Y and Z, for printing of fibers [358,359,360].

**Figure 32:**
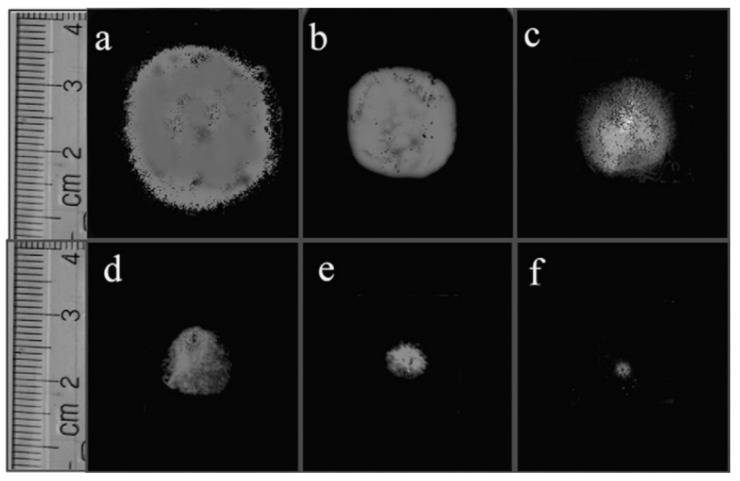
The deposition areas when electrospinning at different parameters. The applied voltage and electrode-to-collector distance are: (**a**) 10 kV and L = 2.6 cm; (**b**) 10 kV and L = 2.1 cm; (**c**) 10 kV and L = 1.7 cm; (**d**) 7 kV and L = 1.2 cm; (**e**) 6 kV and L = 0.8 cm; (**f**) 5 kV and L = 0.5 cm. Reprinted with permission from [105]. Copyright 2008 Elsevier.

**Figure 33:**
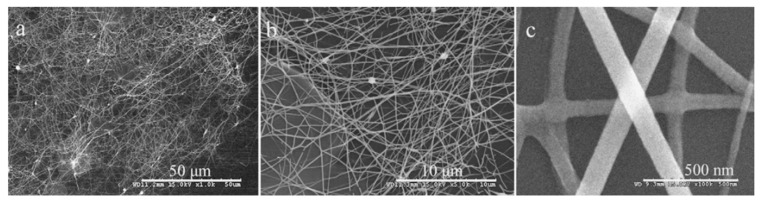
(**a–c**) SEM images of SnO_2_ nanofibers with different magnifications (*T*_an_ = 700 °C). Reprinted with permission from [105]. Copyright 2008 Elsevier.

Zhang et al. [105], by reducing the distance between the spinneret and the collector to 2.6, 2.1 and 1.7 cm, made it possible to reduce the deposition areas to 4, 2.8 and 1.7 cm^2^, respectively (see Figure 32). However, these deposition areas were still much larger than the sensing area of the microhotplate. Only when this distance was decreased to 5 mm did the deposition area decrease to about 2 mm^2^, which was already comparable to the active area of the sensor made on a platform with a micro-heater. Simultaneously with the decrease in the distance, the applied voltage also decreased, which at the minimum distance was 5 kV. Zhang et al. [105] reported that, as a result of the optimization of the electrospinning process of the PVA/SnCl_4_·5H_2_O solution, they were able to obtain SnO_2_ nanofibers with an average diameter of ~100 nm (see Figure 33) and construct, on their basis, a sensor with a large response to 10 ppm ethanol (~4.5), a low detection limit (10 ppb) and fast response/recovery processes (t < 14 s). This is a good achievement; however, the proposed approach was not further developed in the development of gas sensors. In addition, the fibers, formed using near-field methods, are generally much thicker than far-field electrospinning fibers, and the complexity of the device limits their use for mass production [358,359,360]; however, using an automated X-Y translational motion stage combined with near-field electrospinning, it would be possible to develop a technology for the formation of gas-sensitive layers similar to injection printing. For other purposes, this approach has already been implemented in [361,362,363]. However, it must be recognized that the performance of such a technology would be incomparable with the capabilities of injection printing.

There are also other approaches to the patterning of nanofibers in the process of forming a nanofiber mat [352], such as direct writing using melt electrospinning [364], selective photo-crosslinking of electrospun nanofibers containing a photoinitiator to generate a patterned mat [365] and localized removal of nanofibers using a laser, UV or a solvent [366,367,368]. However, all of these techniques are designed for the patterning of polymer nanofiber mats.

- **Seventh**, the low production rate is another important disadvantage of this method. Therefore, the major task after success in laboratories is to optimize the electrospinning process in order to increase productivity.

**Figure 34:**
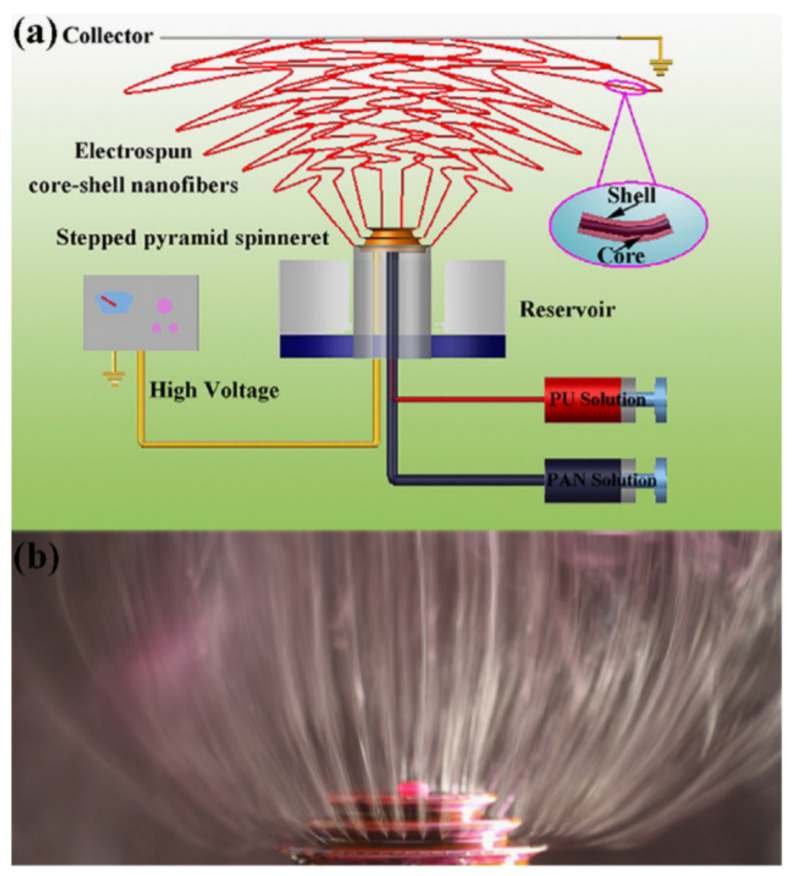
(**a**) Scheme of free coaxial electrospinning apparatus using a stepped pyramid spinneret; (**b**) a picture of coaxial jets in electrospinning process. Reprinted with permission from [370]. Copyright 2014 Elsevier.

Wang et al. [369] believe that a modification of the injection system, such as the introduction of multi-spinneret components that allows parallel multiprocessing and the development of free surface electrospinning methods, can solve the problem of increasing production volumes. A schematic diagram of this modified free surface coaxial electrospinning setup is shown in Figure 34, which contains five main components: a high-voltage direct current power supply, a stepped pyramid spinneret, a Teflon solution reservoir, two pumps and a grounded collector [370]. A stepped pyramid spinneret was used as the electrospinning generator. Indeed, the introduction of these developments made it possible to increase the rate of formation of nanofibers by more than 250 times [352,369]. However, even with the implementation of these modifications, the speed of fiber production using electrospinning technology will be much lower compared to other existing technologies for forming a sensitive layer used in thick-film technology. In addition, this technology is not suitable for forming nanofiber mats in small areas of the gas sensor platform, as required by the technology of manufacturing gas sensors.

- **Eighth**, electrospun nanofibers are mostly randomly oriented (see Figure 26, Figure 28 and Figure 33), which significantly limits the ability to obtain the required repeatability of the final structures of nanofiber mats [371]. In this case, due to the arbitrary position of nanofibers on the surface of the substrates, the fewer fibers there are in the coating, the lower the repeatability of the fiber mat structure.

**Figure 35:**
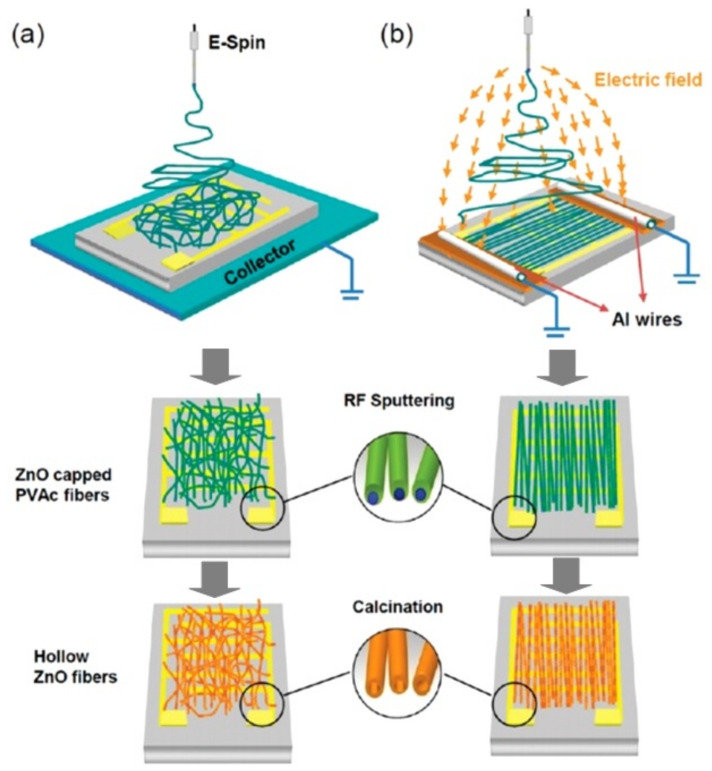
Schematic diagram illustrating the fabrication procedure of an array of (**a**) nonaligned and (**b**) quasi-aligned hollow ZnO fibers. Reprinted with permission from [138]. Copyright 2009 ACS.

Researchers are trying to overcome this limitation with new methods of electrospinning [90] which give better control over the electrospun nanofiber orientation. For example, Choi et al. [138] suggested using aligned nanofibers to fabricate a gas-sensitive layer. To align the fibers parallel to each other along some axis, two strips of aluminum wires were placed along opposite edges of the substrate and connected to the ground terminal of the power supply, which created an electric field between the nozzle and the substrate during the electrospinning processes. This imposed a directional distribution of electric field lines between the nozzle and the aluminum wires, as shown in Figure 35, making it easier to align the fiber segments along the aluminum wires [101]. This technique was discussed earlier. Undoubtedly, the use of aligned nanofibers should increase the repeatability of the structure of the gas-sensitive layer and hence improve the reproducibility of the sensor parameters. Choi et al. [138] also found that NO_2_ sensors with aligned ZnO nanofibers had increased sensitivity compared to sensors using nonaligned ZnO nanofibers. They suggested that this effect is due to the specificity of the current flow in structures with aligned nanofibers.

- **Ninth**, and most importantly, electrospinning technology is incompatible with traditional mass production processes.

Dong et al. [192] believe that this problem can be solved by eliminating the use of long fibers. They proposed fragmenting SnO_2_:Pt nanofibers into smaller pieces (several micrometers long) by ultrasonication in isopropanol and then using them as ink for printing on a microplatform with finger Au electrodes and a Pt micro-heater. The fabricated gas sensors operated at a power below 36 mW at 300 °C. At this temperature, the sensor response to 20 ppm H_2_S exceeded 5000. H_2_S sensors based on SnO_2_:CuO nanofibers were manufactured in the same way [194].

**Figure 36:**
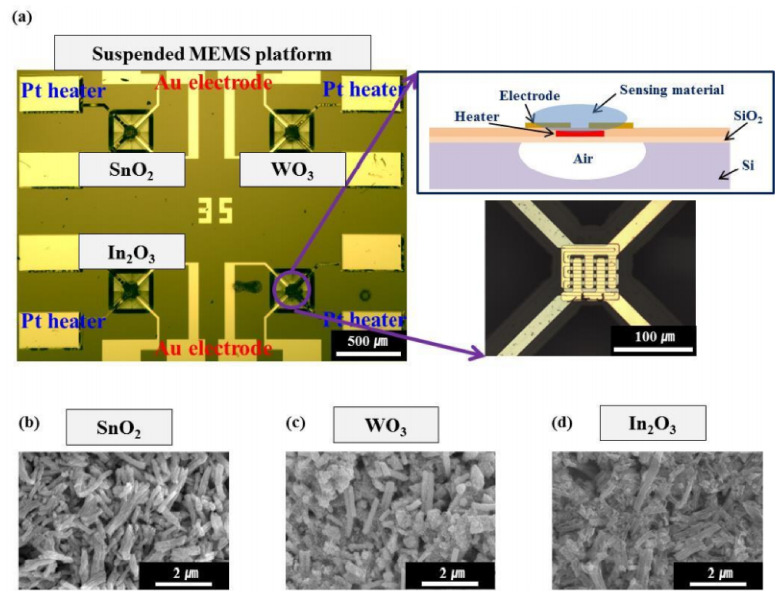
(**a**) MEMS gas sensor array fabricated by EHD printing of SnO_2_, WO_3_ and In_2_O_3_ nanofibers for low power consumption. The platform size is 3.5 × 3.5 mm. The configuration and SEM image of the individual element are also given here. (**b**–**d**) SEM images of nanofiber materials integrated on the suspended MEMS platform by EHD printing. Reprinted with permission from [372]. Copyright 2017 Elsevier.

**Figure 37:**
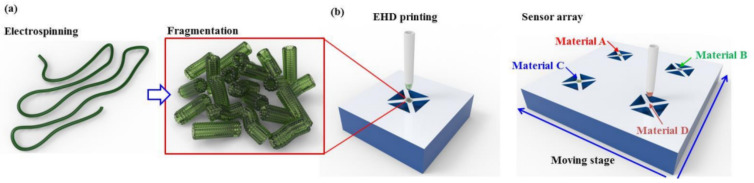
Fabrication of highly integrated gas sensor array by EHD printing of electrospun nanofibers: (**a**) metal oxide nanofiber fragments are prepared by electrospinning and fragmentation process (e.g., ultrasonication); (**b**) individual gas sensor and highly integrated gas sensor array were fabricated by micro-patterning of heterogeneous metal oxide nanofibers via sequential or parallel EHD printing process. Reprinted with permission from [372]. Copyright 2017 Elsevier.

Kang et al. [372] also demonstrated the effectiveness of the proposed technology for the manufacture of gas sensors. The ink prepared on the base of fragmented nanofibers was used for electrohydrodynamic (EHD) printing. This technique was tested for four metal oxides: SnO_2_, WO_3_, In_2_O_3_ and NiO. Each metal oxide nanofiber was mixed with ethanol, and then ultrasonication process was conducted for an hour. After ultrasonication, the ethanol in the nanofiber solution was evaporated in a convection oven at 70 °C for 9 h. The dried electrospun fibers were finally dispersed in alpha-terpineol or ethyleneglycol solvents with a 15 wt% concentration. After fragmentation, the length of SnO_2_, WO_3_, In_2_O_3_ and NiO nanofibers, used to fabricate gas sensors, was ~60–70 μm, 40–60 μm, 55–70 μm and 60–70 μm, respectively. For the manufacture of gas sensors, a platform with a micro-heater was used (Figure 36). This process is illustrated in Figure 37. The active area of each gas sensor was smaller than 100 × 100 μm^2^. Testing of manufactured sensors for sensitivity to NO_2_, H_2_S and CO showed that all sensors could detect NO_2_ (0.1 ppm), H_2_S (1 ppm) and CO (20 ppm).

Yan et al. [373], Du et al. [327] and Zhang et al. [139] used an even simpler technique for the manufacture of sensors. After the formation of metal oxide nanofibers and their calcination at 550–600 °C, Yan et al. [373] and Du et al. [327] made a paste from these fibers using ethanol as the solvent. Subsequently, the pasts were applied on ceramic tubes with a pair of gold electrodes. Lim et al. [160] also used this approach to fabricate In_2_O_3_ nanofiber-based gas sensors. The sensor was fabricated by dropping In_2_O_3_ gel on a sapphire substrate with pre-patterned Pt electrodes. The nanofiber-based gel, in addition to In_2_O_3_ nanofibers, contained deionized water and agate mortar. Unfortunately, the authors did not report what changes occurred in the nanofibers as a result of such processing. Regarding the parameters of these sensors, the sensors fabricated by Yan et al. [373] had a high sensitivity to ethanol. At an ethanol concentration of 100 ppm, the sensor response at 280 °C reached 40.

It must be recognized that the approach suggested by Dong et al. [192], Kang et al. [372] and Yan et al. [373] allows the fabrication of nanofiber-based sensors using thick-film technology compatible with mass production. However, this technology, unfortunately, does not solve the other problems of electrospinning described earlier. In addition, a new technological operation, such as ultrasonication, which requires control, is added. Rejecting the traditional configuration of nanofiber-based membranes, we also lose the main advantage of nanofiber-based sensors, namely, good performance. For example, sensors based on fragmented In_2_O_3_ and NiO nanofibers that showed a faster response and recovery, at a temperature of 300 °C, had a response time/recovery time of 24 s/92 s and 32 s/43 s, respectively. For comparison, In_2_O_3_ nanofiber-based ethanol sensors, made from aligned nanofibers based on the standard nanofiber-based sensor manufacturing approach, had response and recovery times of 1 s and 3 s, respectively [100].

## 5. Summary

The analysis carried out in Part 1 and Part 2 of this article showed that electrospinning technology really presents great opportunities for the formation of gas-sensitive materials with a unique combination of parameters. Electrospinning makes it possible to form a gas-sensitive matrix from small crystallites while maintaining a very high gas permeability of the matrix due to the ultra-high porosity of the structure. This, on the one hand, provides an ultra-high sensitivity of the sensors under optimal conditions and, on the other hand, guarantees a fast response and recovery, since such a structure has no diffusion restrictions for gas penetration into the gas-sensitive matrix. However, it should be noted that this is only possible if the sensor response is limited by gas diffusion in the gas-sensitive matrix. If the kinetics of the sensor response is controlled by the kinetics of surface processes, then in this case, there will be no improvement in the kinetics of the sensor response.

However, it must be admitted that sensors manufactured using traditional technology, with appropriate optimization of the structure and composition of the gas-sensitive layer, can have the same parameters and, in some cases, even exceed the parameters reported for the best samples of nanofiber-based gas sensors [197,251,297,374,375,376]. This means that no breakthrough has been achieved in the development of gas sensors based on the use of electrospinning technology, which could manifest itself in a radical improvement in the parameters of devices in comparison with conventional technology. Unfortunately, the fulfillment of this condition is one of the prerequisites for the implementation of new technologies. If the performances of the devices being developed change insignificantly, then it makes no sense to bear the high costs associated with the implementation and development of a new technological process. This is exactly the situation in the sensor market, where there are still ceramic sensors, the technology of which was developed more than 40 years ago. If we add to this the technological difficulties that arise when organizing the mass production of nanofiber-based gas sensors, then we can confidently state that nanofiber-based sensors cannot yet compete with cheaper devices manufactured using traditional thick-film and thin-film technologies. It is important to note here that this conclusion applies only to conductometric gas sensors and does not apply to other areas where electrospun nanofibers can be used with great success [11,344,352,369,377,378,379].

Based on the above, we can conclude that electrospinning technology, despite some advantages, is unlikely to be used in the near future in the development of gas sensors designed for the market. At the same time, in the development of sensors designed for specific applications such as the security sector and the fight against terrorism, where ultra-high sensitivity is required with a quick response, this technology can find application. In this area, manufacturability and price fade into the background. Using electrospinning technology to find new gas-sensitive materials with specific multi-functional properties, combining unique structural, electrical and physical properties, is also of interest and promise in the future in the development of new-generation sensors. The ability to form solid, hollow and core–shell fibers with a controlled structure and composition offers great opportunities for this. It is only necessary to find technological solutions to introduce this technology into mass production.

## Figures and Tables

**Table 4:** Influence of doping with Ce (6 mol%) on the parameters of SnO_2_ nanofibers and their conductometric response to ethanol (50 ppm) at *T*_oper_ = 250 °C.

Sample	Fiber Diameter, nm	Crystallite Size, nm	Surface Area, m^2^/g	Response
SnO_2_	~234	41	16.7	~20
SnO_2_:Ce	~127	27	35	~260

*Source*: data extracted from [229].

**Table 5:** Influence of additives (in oxide form) in metal oxide matrix on structural and gas sensing characteristics of SnO_2_- and In_2_O_3_-based sensors. Influence on the electrophysical characteristics, i.e., acceptor or donor behavior of the additives, is not considered.

Additive	Effect	Nature
Al_2_O_3_; SiO_2_	Increases sensor response; improves thermal stability	Decrease in crystallite size; decrease in the area of intergrain contacts; increase in porosity
Ag (Ag_2_O); Cu (Cu_2_O)	Increases response to H2S, SO2	Two-phase system; phase transformations during gas detection
Fe (Fe_2_O_3_)	Increases response to alcohols	Change in oxidation state
Ga(Ga_2_O_3_); Zn(ZnO)	Increases sensor response	Decrease in crystallite size; increase in porosity
P, B	Improves selectivity	Creation of new phase
Se	Increases sensor response	Increase in porosity
Ca; K; Rb; Mg	Increases sensor response; improves thermal stability	Decrease in crystallite size
La; Ba; Y; Ce	Improves thermal stability; increases sensor response	Stabilization of crystallite size (creation of new phases); decrease in crystallite size
Transition MOXs: Co; Mn; Sr; Ni	Increases sensor response; improves selectivity	Catalytic effect; change in electron concentration; change in A/D parameters; change in crystallite size

*Source*: Reprinted with permission from [60]. Copyright 2017 Elsevier.

**Table 8:** Influence of oxygen plasma treatment on the parameters of In_2_O_3_ fibers, and conductometric response to acetone.

Sample	Fiber Diameter, nm	Crystallite Size, nm	Surface Area, m^2^/g	*T*_oper_, °C	S (100 ppm)	*τ*_res,_ s
In_2_O_3_	~100	35	18.2	275	~7	23
In_2_O_3_-O_2_	~170	23	32.5	275	~20	27

*Source*: data extracted from [327].

## Data Availability

All the data are reported in the paper directly.
